# Human Fetal Scalp Dermal Papilla Enriched Genes and the Role of R-Spondin-1 in the Restoration of Hair Neogenesis in Adult Mouse Cells

**DOI:** 10.3389/fcell.2020.583434

**Published:** 2020-11-26

**Authors:** Erin L. Weber, Yung-Chih Lai, Mingxing Lei, Ting-Xin Jiang, Cheng-Ming Chuong

**Affiliations:** ^1^Department of Pathology, University of Southern California, Los Angeles, CA, United States; ^2^Division of Plastic Surgery, Indiana University School of Medicine, Indianapolis, IN, United States; ^3^Integrative Stem Cell Center, China Medical University Hospital, China Medical University, Taichung, Taiwan; ^4^111 Project Laboratory of Biomechanics and Tissue Repair, College of Bioengineering, Chongqing University, Chongqing, China

**Keywords:** hair induction, follicle neogenesis, skin reconstitution, dermal papilla, human

## Abstract

Much remains unknown about the regulatory networks which govern the dermal papilla’s (DP) ability to induce hair follicle neogenesis, a capacity which decreases greatly with age. To further define the core genes which characterize the DP cell and to identify pathways prominent in DP cells with greater hair inductive capacity, comparative transcriptome analyses of human fetal and adult dermal follicular cells were performed. 121 genes were significantly upregulated in fetal DP cells in comparison to both fetal dermal sheath cup (DSC) cells and interfollicular dermal (IFD) populations. Comparison of the set of enriched human fetal DP genes with human adult DP, newborn mouse DP, and embryonic mouse dermal condensation (DC) cells revealed differences in the expression of Wnt/β-catenin, Shh, FGF, BMP, and Notch signaling pathways. We chose R-spondin-1, a Wnt agonist, for functional verification and show that exogenous administration restores hair follicle neogenesis from adult mouse cells in skin reconstitution assays. To explore upstream regulators of fetal DP gene expression, we identified twenty-nine transcription factors which are upregulated in human fetal DP cells compared to adult DP cells. Of these, seven transcription factor binding motifs were significantly enriched in the candidate promoter regions of genes differentially expressed between fetal and adult DP cells, suggesting a potential role in the regulatory network which confers the fetal DP phenotype and a possible relationship to the induction of follicle neogenesis.

## Introduction

The DP, though distinctly different from the DC and primarily responsible for cyclical regeneration of the mature follicle, retains the follicle-inducing property of its DC progenitor. Many studies have demonstrated the capacity of the whole DP or cultured DP cells to induce new hair follicle formation in hairless epidermis (Jahoda, 1992; Jahoda et al., 1993). However, the efficiency of follicle neogenesis varies greatly by organism, follicle type, and age, with rat vibrissae and newborn mouse pelage DPs exhibiting much higher rates of follicle neogenesis than adult mouse or human DPs (Jahoda et al., 2001; Higgins et al., 2013; Thangapazham et al., 2013). Clinically, the efficiency of human adult DP inductivity is ineffectively low to be useful for the treatment of alopecia and requires the harvest of functional follicles, making it no less invasive or limiting than the current gold standard of follicular transplantation. If existing adult DP cells could be expanded in culture and reprogrammed to possess higher inductive capacity akin to the fetal DC or DP, transplantation of such cells may offer a new treatment for androgenetic alopecia or skin injury (Chueh et al., 2013; Higgins and Christiano, 2014).

With this in mind, we investigated the gene expression profile of the human fetal DP, a structure which is more anatomically distinct than the DC, and, through comparative transcriptomics, determined differences in gene expression between fetal and adult DP cells. We identified 121 enriched human fetal DP genes and a subset of transcription factors upregulated in fetal DP cells compared to adult DP cells which may regulate the developmentally active fetal DP. Our previous work on the differences between murine neonatal and adult hair follicle neogenesis identified important signaling pathways and described robust *in vitro* and *in vivo* hair reconstitution assays for the examination of key factors in follicle development (Lei et al., 2017a). In this analysis of human fetal DP cells, the R-spondins, a family of *Wnt* agonists, were differentially upregulated and the exogenous administration of R-spondin-1 rescued hair follicle neogenesis in adult mouse reconstitution assays.

## Materials and Methods

### Human Tissue

Two adult, non-balding scalp specimens were obtained through the National Disease Research Interchange (Philadelphia) from deceased 36- and 54-year old males. Two fetal scalp specimens were obtained through Novogenix, Inc. (Los Angeles) from second trimester fetuses electively aborted at developmental ages 16 and 17 weeks. Procurement protocols for both organizations involved appropriate informed consent for donated tissues.

### Isolation of Cell Populations

**FIGURE 1:**
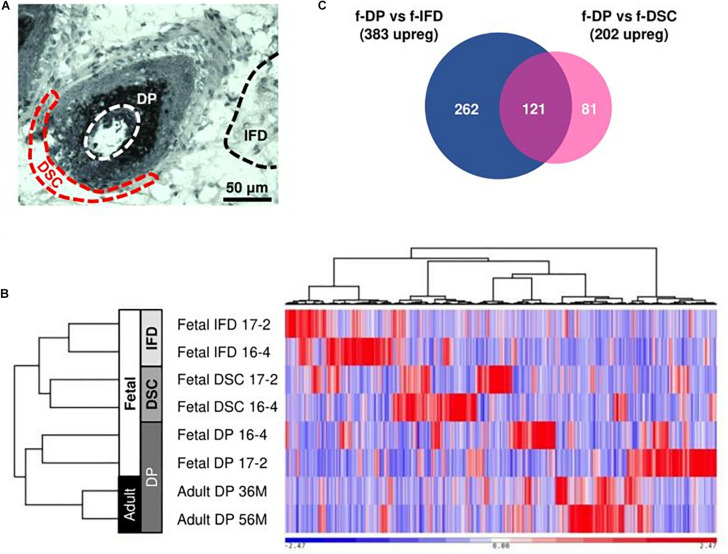
Low-input RNA-Seq analysis of human scalp. **(A)** DP, DSC, and IFD cells were manually harvested from frozen sections of human scalp tissue. **(B)** Hierarchical clustering of RNA-Seq samples demonstrated clustering of similar cell types despite intersample variation. **(C)** 121 enriched fetal DP genes were identified from comparison of the fetal DP transcriptome with fetal DSC and IFD transcriptomes.

Frozen sections were stained with the Arcturus Histogene Kit. IFD regions and DP and DSC cells from anagen-phase follicles were dissected using pulled glass capillary tubes under magnification (Figure 1A). RNA was extracted with the Arcturus PicoPure RNA Isolation Kit. cDNA was amplified from 500 pg of RNA per sample using the Nugen Ovation RNA-Seq System V2 and fragmented into 300bp segments using a Covaris sonicator. RNA-Seq libraries were constructed from 80 to 100 ng of cDNA with the Nugen Ovation Ultralow System V2. Sample concentration and quality was assessed with the Agilent Bioanalyzer.

### RNA-Seq Analysis

More than 100 million 75-bp single-end reads were generated for each RNA-Seq sample using an Illumina NextSeq 500 sequencer. QualiMap2 was used to measure RNA degradation and genomic DNA contamination (Okonechnikov et al., 2015). For secondary RNA-Seq analysis, the human hg19 assembly, including unplaced and unlocalized scaffolds and RefGene annotation, was downloaded from the UCSC Genome Browser on 2016.2.6 (Speir et al., 2016). Low quality bases were trimmed based on the Phred quality score (>20) from both the 5′- and 3′-ends. After trimming, reads <50bp or with ambiguous bases were discarded. Alignment, quantification, normalization, and differential expression analysis were performed by STAR 2.4.1d (Dobin et al., 2013) through Partek Flow (Partek Inc.), htseq-count 0.6.0 (Anders et al., 2014), TMM (Robinson and Oshlack, 2010), and edgeR 3.10.5 (Robinson and Smyth, 2008), respectively. Genes with count-per-million values >1 in at least two samples were retained. The false discovery rate (FDR) was set at <0.05. Principal component analysis, Ward’s hierarchical method (Ward, 1963), and Venn diagrams were performed with Partek Genomics Suite 6.16 (Partek Inc.). Pathway enrichment analysis using Fisher’s exact test and Upstream Regulator Analysis were performed with QIAGEN’s Ingenuity^®^ Pathway Analysis. IPA was also used to build a regulatory network for hair follicle regenerative potential between fetal and adult DP cells. Transcription factor binding sites (TFBSs) within candidate promoter regions were predicted by FMatch (Kel et al., 2003) based on the TRANSFAC database (Matys et al., 2006). Candidate promoter sequences were defined as 1,000bp upstream and 100bp downstream of transcription start sites. The 882 non-differentially expressed genes with the largest FDR values were used as the background set. The specified cut-offs were selected as the minimum of the sum of both error rates (minSUM) for the matrix similarity score and *p*-value <0.01 for TFBSs enrichment.

### Murine Hair Reconstitution Assay

Dorsal skin from 2 month old K14-H2B-GFP transgenic mice was incubated in 0.25% trypsin solution at 4°C overnight. The epidermis was dissociated into a single-cell suspension by repeated pipette titration and passage through a 70 μm filter. A single-cell dermal suspension was achieved by incubation in 0.35% collagenase I solution (Worthington) for 30 min at 37°C, followed by the addition of DNAse I (Qiagen) and 5 min incubation at room temperature. Medium containing 10% fetal bovine serum (FBS) was added and the dermal cells were dissociated by repeated pipette titration and filtration through a 40 μm filter. Epidermal and dermal cells were recombined in a 1:9 ratio in DMEM/F12 culture medium (1:1, Gibco) with 10% FBS (Gibco) and plated drop-wise on a 6-well culture insert submerged in culture medium. Cultures were incubated at 37°C and 5% CO_2_. The following factors were added to the culture medium: 5 μM protein kinase C inhibitor (Bisindolyl maleimide I, Cayman Chemical, day 0–6), 25 ng/ml R-spondin-1 (R&D Systems, day 0–2), 10 ng/ml Wnt3a (R&D Systems, day 1–4), and 10 ng/ml MMP14 (R&D Systems, day 3–6). On day 6, cell culture inserts were sutured droplet-side down to full-thickness dermal wounds on nude mice. Hair growth was observed over 6 weeks. Culture droplets and skin biopsies were stained with propidium iodide and imaged with a confocal microscope. Epidermal cells from transgenic mice were detected by GFP fluorescence.

## Results

Hair follicle DP cells from different body regions or developmental ages display varying patterns of gene expression (Chen et al., 2015). We employed the following strategy to identify important human fetal scalp DP genes. To identify genes unique to the fetal DP, we dissected DP cells from mature, anagen-phase, fetal follicles and compared them with adjacent DSC cells, known to be transcriptionally distinct but developmentally related to DP cells, and IFD cells, comprised of a heterogeneous mixture of non-follicular fibroblasts. To identify core genes shared by DP cells across different organisms, ages, and follicle types, we compared the fetal scalp DP transcriptome with human adult DP, newborn mouse DP, and embryonic mouse DC datasets. To elucidate signaling pathways which may confer fetal DP cells with a greater ability to induce follicle neogenesis, we compared transcriptomes from human fetal and adult anagen-phase DP cells. We identified key transcription factor binding motifs in promoter regions upstream of genes differentially expressed in fetal compared to adult DP cells to determine potential upstream regulators and a foundation for multiple downstream signaling networks. Finally, we employed skin reconstitution assays using adult murine skin cells, which inefficiently induce follicle neogenesis, to demonstrate that R-spondin-1, a member of one of the gene families upregulated in human fetal DP cells, can restore the ability to form new follicles.

### Identification of Human Fetal Dermal Papilla Enriched Genes

Cluster analysis of 16,623 expressed genes demonstrated appropriate grouping of biological replicates, with adult and fetal DP cells clustering distinctly from DSC and IFD cells (Figure 1B). We must note that, due to the necessary protocols surrounding ethical acquisition of human tissue, all libraries were derived from moderately degraded, low quantity samples amplified by random primers. Consequently, biological variation within replicates was apparent (Figure 1B). The 3′ enrichment of sequencing reads reflected RNA degradation (Supplementary Figure 1 and Supplementary Table 1) and an increased proportion of intergenic and intronic reads indicated genomic DNA contamination detected by random primers (Supplementary Figure 1 and Supplementary Table 1). Nonetheless, due to the lack of better human tissue acquisition methods, the RNA-Seq libraries were acceptable for further comparative analysis.

**FIGURE 2:**
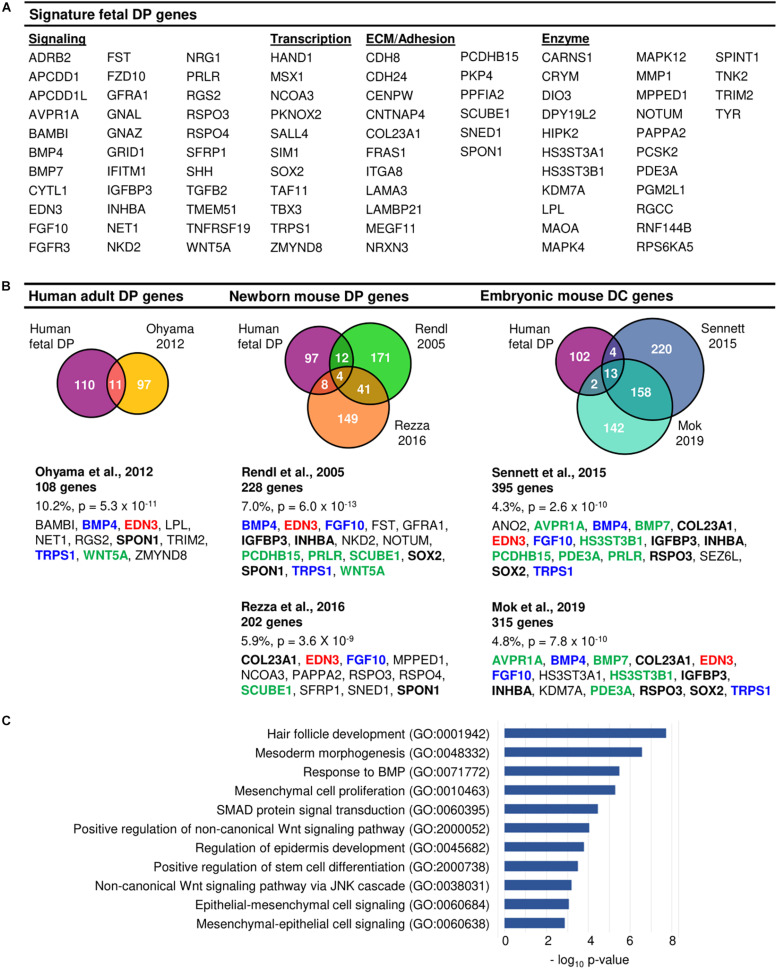
Molecular enrichment for the human fetal DP. **(A)** Enriched human fetal DP genes. **(B)** Comparison of the human fetal DP enriched gene profile with previously published human adult DP, newborn mouse DP, and embryonic mouse DC signature gene sets demonstrated significant overlap. Genes listed in red were present in 6 datasets, genes in blue in 5, genes in bold black in 4, and genes in green in 3. **(C)** Gene ontology analysis of the human fetal DP enriched genes confirmed the prevalence of pathways known to be important for hair follicle development.

1,035 differentially expressed genes (383 upregulated, 652 downregulated) were identified between fetal DP and IFD cells and 564 differentially expressed genes (202 upregulated, 362 downregulated) were identified between fetal DP and DSC cells. The DP expression profile differed more markedly from IFD cells than DSC (DP vs. IFD: *r* = 0.42 ± 0.02, DP vs. DSC: *r* = 0.66 ± 0.04, *p* < 0.001). 121 genes were upregulated in fetal DP cells compared to DSC and IFD cells and were designated enriched human fetal DP genes (Figures 1C, 2A and Supplementary Table 2). Compared with published datasets, the enriched human fetal DP genes identified in this study significantly overlapped with human adult DP signature genes identified by Ohyama et al. (2012) (11/108, *p* = 5.3 × 10^–11^), neonatal mouse DP signature genes identified by Rendl et al. (2005) (16/228, *p* = 6.0 × 10^–13^) and Rezza et al. (2016) (12/202, *p* = 3.6 × 10^–9^), and embryonic mouse DC genes identified by Sennett et al., 2015 (17/395, *p* = 2.6 × 10^–10^) and Mok et al., 2019 (15/315, *p* = 7.8 × 10^–10^) (Figure 2B). *Edn3* was present in all six datasets; *Bmp4, Fgf10, and Trps1* were present in five; and *Col23a1, Igfbp3, Inhba, Rspo3, Spon1, and Sox2* were present in four, suggesting that expression may play an important role in the DP during hair follicle neogenesis, development, or cycling. Gene ontology analysis of the enriched human fetal DP genes confirmed multiple pathways known to be important for hair follicle development, including BMP, Wnt, and epithelial-mesenchymal signaling (Figure 2C).

### Differential Gene Expression Between Human Fetal and Adult Dermal Papillae

**FIGURE 3:**
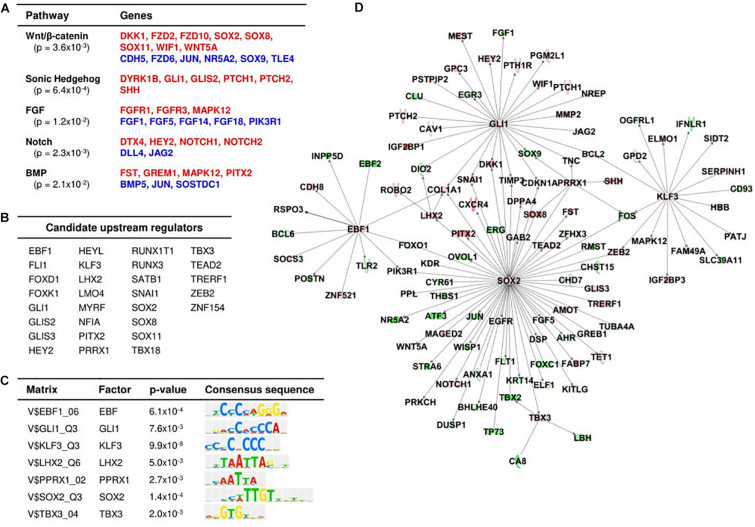
Differential gene expression in human fetal DP compared to adult DP cells. **(A)** Significantly enriched pathways of differentially expressed genes between fetal DP and adult DP. The red and blue highlighted genes indicate upregulation and downregulation, respectively, in fetal DP cells compared to adult DP cells. **(B)** Candidate upstream transcriptional regulators upregulated in fetal versus adult DP. **(C)** Significantly enriched transcription factor binding motifs in the promoter sequences of differentially expressed genes between fetal and adult DP. **(D)** Predicated regulatory network of fetal DP gene expression. Red and green highlighting denotes upregulation and downregulation, respectively, in fetal DP compared to adult DP cells.

822 differentially expressed genes (356 upregulated, 466 downregulated) were identified between fetal and adult DP cells (Supplementary Table 3). Validation of fetal RNAseq data was performed through immunofluorescent staining of fetal and adult scalp with genes present in the enriched fetal DP dataset, upregulated in fetal versus adult DP, and minimally expressed in DSC and IFD cells (Supplementary Figure 2). Hair follicle-associated pathways were significantly enriched, including Wnt/β-catenin (*p* = 0.004), sonic hedgehog (*p* = 0.0006), FGF (*p* = 0.01), Notch (*p* = 0.002), and BMP signaling (*p* = 0.02) (Figure 3A). Twenty-nine transcription factors were upregulated in fetal compared to adult DP cells (Figure 3B). Enrichment analysis of TFBSs within candidate promoter regions of genes differentially expressed between fetal and adult DP identified seven significantly enriched transcription factors, suggesting a role as important upstream regulators for the fetal DP phenotype (Figure 3C). Using the IPA database, we constructed a working hypothesis of a regulatory network with 110 genes differentially expressed between fetal and adult DP cells (Figure 3D). Within this model, SOX2 and GLI1, two of the seven enriched TFBSs, interact with a high proportion of the enriched fetal DP genes and are promising hubs for regulation of downstream differential gene expression. SOX2 was predicted as the top transcriptional regulator (*p* = 2.88 × 10^–15^).

### R-Spondin-1 Is Important for the Restoration of Hair Follicle Neogenesis From Adult Mouse Skin Cells

Wnt signaling is critical for both developmental hair follicle neogenesis and temporal hair cycling. The nuances of such signaling, which distinguish the two types of hair regeneration, are less clear. R-spondins are Wnt agonists which are important for the development of numerous organs, as well as the maintenance of stem cells and epithelial regeneration in the intestine (De Lau et al., 2012; Neufeld et al., 2012; Cambler et al., 2014; Harnack et al., 2019). R-spondin-3 (*Rspo3*) and R-spondin-4 (*Rspo4*) were present in the human fetal DP enriched gene set while R-spondin-1 (*Rspo1*) and *Rspo3* were upregulated in fetal, compared to adult, DP cells. *Rspo2* and *Rspo4* were more highly expressed in adult DP cells. *Rspo3* has been identified as a signature gene in some transcriptional analyses of embryonic and newborn mouse DP (Sennett et al., 2015; Rezza et al., 2016) but there are no reports of defects in follicular development or hair cycling associated with *Rspo3* mutation. *Rspo1* is upregulated in mice in late telogen and early anagen and intradermal injection into telogen skin induces early transition into the anagen phase through activation of epidermal follicle stem cells (Li et al., 2016).

**FIGURE 4:**
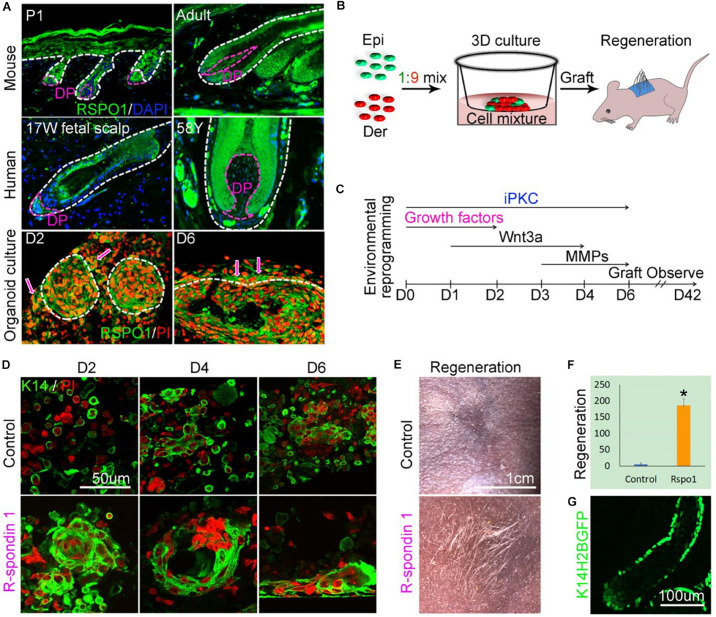
Recovery of follicle neogenesis from adult mouse epidermal and dermal cells through environmental reprogramming. **(A)** Characterization of RSPO1 expression in mouse and human samples. **(B)** Experimental design of the murine skin organoid culture, followed by grafting of cells to dermal wounds on nude mice. **(C)** Environmental reprogramming through the sequential administration of exogenous factors to culture medium from days 0 – 6 (D0 – D6). **(D,E)** Adult epidermal and dermal cells treated with *RSPO1, iPKC, WNT3A*, and *MMP14* demonstrate keratinocyte aggregation (day 2), cyst formation (day 4), planar epidermis formation (day 6), and mature hair follicle formation with hair shafts (D42) compared to untreated (control) adult cells which are unable to organize. Green immunofluorescence reflects K14 positive cells, nuclei are labeled with propidium iodide in red. Clusters of nuclei appear as larger red areas. **(F)** Quantification of hair regeneration, *p* < 0.05. **(G)** K14-H2BGFP immunofluorescence demonstrates derivation of hair follicle epidermal cells from donor adult cells.

*Rspo1* is expressed in both epithelial and DP cells of the murine hair follicle (Harshuk-Shabso et al., 2020). By immunofluorescent staining, we also observed that *Rspo1* is expressed in the epithelial and DP cells of both mouse and human hair follicles. *Rspo1* expression is decreased in the DP of adult mouse and human hair follicles, compared to newborn mouse and fetal scalp hair follicles (Figure 4A). *Rspo1* is also expressed in the newborn mouse epidermal and dermal cells subjected to skin organoid culture. In particular, *Rspo1* is more highly expressed in dermal cells adjacent to epidermal aggregates in day 6 cultures.

Without a robust human hair induction or hair cycling assay at hand, we used a murine skin organoid culture system to test the function of R-spondins during human follicle neogenesis, as it permits the study of dissociated epidermal and dermal cells as they undergo morphological transitions through early skin and appendage development (Figures 4B,D; Lei et al., 2017a). After six days in three-dimensional (3D) culture, newborn mouse epidermal and dermal cells form mature, hair-producing follicles upon grafting to a nude mouse (Figures 4B,E). Adult mouse skin cells, however, have lost the ability to self-organize during skin organoid culture and do not generate hair follicles after grafting (Figures 4D,E). Our previous work defined a combination of exogenous growth factors which, when added to the 3D culture medium, stimulated adult cells to progress through aggregation steps similar to newborn mouse cells (Figure 4C; Lei et al., 2017a).

To avoid bias introduced by species-specific variation, we chose to investigate the role of *Rspo1* in murine hair follicle neogenesis, as a role in the hair cycling is known (Neufeld et al., 2012; Li et al., 2016). The addition of RSPO1 from day 0–2 resulted in larger cell aggregates at day 2. The aggregates progressed to form larger cysts at day 4 and, consistent with our previous findings, the cysts further coalesced to form planar skin at day 6, after the addition of WNT3A and MMP14 (Figures 4C,D). Significantly more hair-bearing follicles were generated after the RSPO1/WNT3A/MMP14/iPKC treated cells were grafted onto the backs of nude mice, compared to adult controls in the absence of growth factors (Figures 4E,F). GFP fluorescence confirmed that the newly regenerated hair follicles were derived from donor cells (Figure 4G).

## Discussion

The human fetal DP, like the newborn mouse, can induce new hair follicle formation. Much effort has focused on identification of the transcriptional profile of the newborn mouse DP and embryonic mouse DC. As the human hair follicle differs anatomically and functionally from the mouse follicle, an assessment of the transcriptional profile of the human fetal DP has the potential to reveal new genes or pathways important for human follicle neogenesis (Cotsarelis, 2006; Panteleyev, 2016). Here, we identified a set of 121 genes significantly upregulated in human fetal DP cells, compared to fetal DSC and IFD cells. Fourteen genes (*Apcdd1, Bmp4, Bmp7, Fgf10, Fst, Gfra1, Inhba, Prlr, Shh, Sox2, Spint1, Tgf*β*2, Tnfrsf19, and Trps1*) are known to be important for hair follicle morphology and development (St-Jacques et al., 1998; Foitzik et al., 1999; Botchkareva et al., 2000; Brown et al., 2000; Ohuchi et al., 2000; Botchkarev et al., 2001; Craven et al., 2001; Malik et al., 2002; Nakamura et al., 2003; Szabo et al., 2007; Pispa et al., 2008; Driskell et al., 2009; Zouvelou et al., 2009; Shimomura et al., 2010). Comparison with published signature gene datasets for human adult DP, newborn mouse DP, and embryonic mouse DC demonstrated 5–10% shared genes (Rendl et al., 2005; Sennett et al., 2015; Rezza et al., 2016; Mok et al., 2019). While low, this degree of overlap is statistically significant. Transcriptomic differences may arise from differences in species, age, or body region. Only deeply shared genes will be selected in common. Genes which are present in the majority of signature gene datasets are likely to be of fundamental importance for specification of the DP cell.

*Edn3* was present in our human fetal DP enriched dataset as well as the human adult DP, newborn mouse DP, and embryonic mouse DC transcriptomes. EDN3 is upregulated in the DP following epilation but has no known role in follicle neogenesis (Rahmani et al., 2014; Li et al., 2017). Endothelins are most well-known for producing vasoconstriction of smooth muscle cells but also cause contraction of other cell types (Davenport et al., 2016). Recently, Heitman et al. (2020) demonstrated that dermal sheath (DS) cells express many genes characteristic of smooth muscle and DS contraction is responsible for movement of the DP into the superficial dermis during telogen. It is possible that the DP signals DS contraction through Edn3 expression, facilitating DP relocation during hair germ to peg transitions during development. Given the widespread presence in DP transcriptomes, despite variation in species, age, and developmental stage, the role of *Edn3* deserves further investigation.

*Bmp4* and *Trps1* were present in our human fetal DP transcriptome as well as adult human DP, newborn mouse DP, and embryonic mouse DC datasets. *Bmp4* is expressed in the DC and DP of the developing murine follicle and supports DP gene expression in culture (Jang et al., 2010; Chen et al., 2012). BMP expression is downstream of the Wnt/β-catenin pathway and the transcription of many DP genes is predicated upon the expression of BMPs (Rendl et al., 2008; Chen et al., 2012). Redundancy of BMP ligands is likely, as loss of individual BMPs, including BMP4, does not affect follicle neogenesis (Rendl et al., 2008; Nakamura et al., 2013). BMP signaling is believed to play a role in follicular patterning by inhibiting follicle fate in adjacent cells and is required for hair follicle induction by DP cells, through unclear mechanisms (Botchkarev et al., 2001; Rendl et al., 2008; Li et al., 2015). *Trps1* is expressed in the DC and DP of developing mouse follicles and is believed to function as both a transcriptional repressor and activator, increasing the expression of *Wnt* inhibitors (Fantauzzo et al., 2008, 2012). Though the absence of DP *Trps1* expression does not ablate hair follicle development, mutant mice lack vibrissae and exhibit a 50% reduction in pelage follicles (Nakamura et al., 2013).

Comparison of the human fetal DP transcriptome with other published transcriptomes demonstrated a subset of genes, including *Col23a1*, *Fgf10*, *Igfbp3*, *Inhba*, *Rspo3*, and *Sox2*, which were present in neonatal and embryonic, but not adult, DP cells, proposing importance for a stem cell-like state capable of inducing follicle formation. The Wnt/β-catenin pathway plays a critical role in hair follicle neogenesis from the earliest stages and may mediate the first dermal signal for placode formation (Saxena et al., 2019). Thus, a signaling molecule expressed by the DP which stimulates the *Wnt* pathway could be a critical signal for hair follicle induction. R-spondins are *Wnt* activators which function through both the canonical and non-canonical *Wnt*/β-catenin pathways. All four R-spondins are expressed in the mouse anagen DP and injection of *Rspo1*, *Rspo2*, and *Rspo3* into adult mouse dermis induces rapid telogen to anagen transition and activation of epidermal follicular stem cells (Li et al., 2016; Smith et al., 2016; Hagner et al., 2020; Harshuk-Shabso et al., 2020). Much less is known about the role of R-spondins in hair follicle development. *Rspo3* can activate the *Wnt*/planar cell polarity (PCP) pathway, known to play a role in hair follicle cell differentiation during development (Ohkawara et al., 2011; Chen and Chuong, 2012). In mice lacking expression of *Fuz*, a PCP gene, hair follicles failed to develop into mature structures (Dai et al., 2011). The absence of *Rspo3* is lethal and conditional knockout yields shorter limbs but no discernible effect on follicle neogenesis (Neufeld et al., 2012). *Rspo1* was also upregulated in human fetal, compared to adult, DP cells but a clear role of *Rspo1* in hair cycling or neogenesis has not been identified to date. For lack of a robust assay using human cells and the inability to conduct cycling or neogenesis assays in humans, we employed murine skin morphogenesis assays to study the effect of R-spondins on follicle development. The addition of *Rspo1* to dissociated adult mouse epidermal and dermal cells, in combination with inhibitors of the PKC pathway, induced epidermal aggregation in culture. With the further addition of WNT and MMP following keratinocyte aggregation, adult mouse epidermal and dermal cells formed functional, mature hair follicles (Lei et al., 2017a). In the absence of these four factors, adult mouse cells are unable to self-organize into any structure resembling a hair follicle. As there is known redundancy in the function of R-spondin genes (Neufeld et al., 2012; Hagner et al., 2020) and *Rspo1* rescues adult hair follicle neogenesis, *Rspo3* is also a promising candidate for the recovery of inductivity in human adult DP cells. In *in vitro* colony-forming assays with human dermal progenitor cells, addition of RSPO2/3 increased proliferation and the number and size of progenitor cell colonies (Hagner et al., 2020).

The functional capacity of hair follicle stem cells depends on the sum of activating and inhibitory signals (Li et al., 2015). While there are core activators and inhibitors shared by all DP cells, DPs of different species, ages, or follicle types may be subjected to alterations in the balance of signals and, thus, possess differing functional capacities, such as the specification of hair filament diameter, temporal and region-specific hair types, or anagen/telogen duration, which affects hair length (Lei et al., 2017b). Here, we sought to identify genes differentially upregulated in fetal DP, compared to adult DP, with the hope of finding transcriptional regulators responsible for fetal DP phenotype and function. Seven transcription factors were enriched in the promoter regions of genes upregulated in the fetal DP. Of these, SOX2 and GLI1 were predicted to be principal regulators of the fetal DP transcriptome. Similarly, *Shh and Wnt* pathways are known to be necessary for hair follicle development and both SOX2 and GLI1 are predicted upstream regulators of numerous *Wnt*-related genes. *Sox2* expression correlates with the trajectory of differentiation from dermal fibroblast to dermal condensate cell fate and is a well-known pluripotency marker (Gupta et al., 2019; Mok et al., 2019). Mutation of *Sox2*, however, does not abolish hair follicle formation but alters the ratio of hair follicle type in mice, suggesting that SOX2 is not the single predominant factor which specifies the fetal DP (Driskell et al., 2009; Nakamura et al., 2013). The downregulation of GLI1, a downstream effector of Shh signaling, in dermal condensates of *Shh* knockout mice is associated with a block in hair follicle development at the DC to DP transition (Chiang et al., 1999). Further evaluation is needed to determine the role of these upstream regulators in the initiation or maintenance of *Shh* and *Wnt* pathways during follicle development.

Limitations of this study include the use of non-clonal human tissue, leading to intersample variation. The acquisition of human tissue is appropriately protected through regulatory procedures, delaying the time from harvest to study and resulting in more sample degradation than murine samples. We extracted RNA from uncultured samples to avoid bias introduced by culturing, which can rapidly affect transcription (Higgins et al., 2013). The trade-off is low quantity RNA samples of lesser quality. However, the transcriptomes of similar tissues clustered together and reflected a hierarchy of similarities consistent with the trajectory of differentiation from fibroblast to DP (Gupta et al., 2019; Mok et al., 2019). The transcriptome of the fetal DP was more similar to the DSC than IFD, contained signaling factors and transcriptional regulators known to play important roles in hair follicle development, and was similar to published murine DP and DC transcriptomes. This work creates a platform for the further study of candidate factors in human hair follicle development. The ability to understand the human signaling and regulatory milieu offers direct avenues for the treatment of alopecia and severe skin injury (Chueh et al., 2013).

## Data Availability Statement

The datasets presented in this study can be found in online repositories. The names of the repository/repositories and accession number(s) can be found below: https://www.ncbi.nlm.nih.gov/geo/, GSE149189.

## Ethics Statement

The animal study was reviewed and approved by the Institutional Animal Care and Use Committee of the University of Southern California.

## Author Contributions

EW, Y-CL, ML, and C-MC designed the study. EW, ML, and T-XJ performed the experiments. EW and Y-CL performed the bioinformatics analysis. EW, Y-CL, and ML wrote the manuscript. All authors were involved in revision of the manuscript and approved the submitted version.

## Conflict of Interest

The authors declare that the research was conducted in the absence of any commercial or financial relationships that could be construed as a potential conflict of interest.

## Abbreviations

DC: dermal condensate
DP: dermal papilla
DS: dermal sheath
DSC: dermal sheath cup
IFD: interfollicular dermis.
