# Supplementary material for: Human Fetal Scalp Dermal Papilla Enriched Genes and the Role of R-Spondin-1 in the Restoration of Hair Neogenesis in Adult Mouse Cells
Source: Front Cell Dev Biol. 2020 Nov 26;8:583434. doi: 10.3389/fcell.2020.583434 (PMC7726222; doi:10.3389/fcell.2020.583434)
Supplement: Supplementary file 1 [file Data_Sheet_1.PDF]

Supplementary Material

A

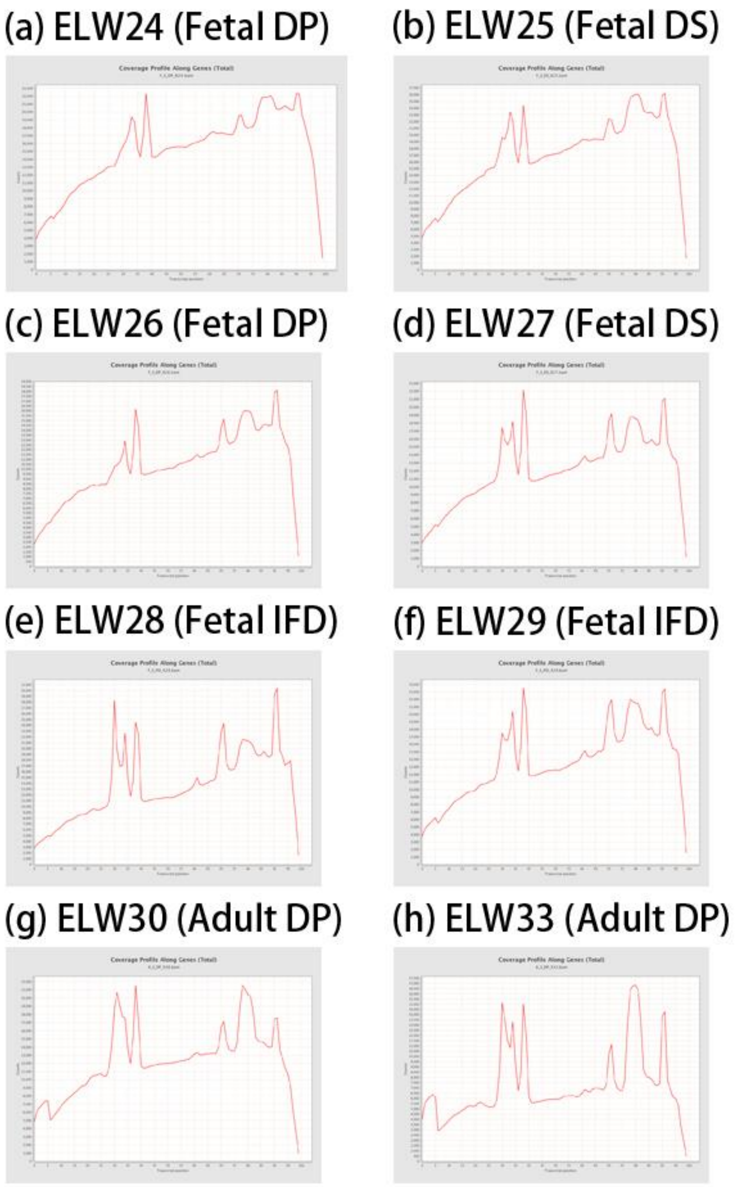

B

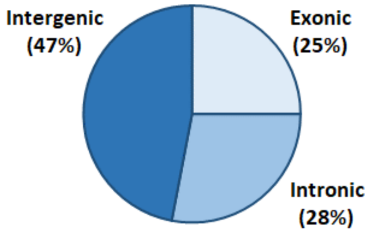

Figure S1. (**A)** Coverage profile along genes. Mean read coverage profiles of 500 highest-expressed genes were demonstrated for sample ELW24 (a), ELW25 (b), ELW26 (c), ELW27 (d), ELW28 (e), ELW29 (f), ELW30 (g), and ELW33 (h), respectively. More enrichment in the 3’ region indicates more degraded samples. **(B)** A high proportion of intergenic and intronic reads reflects DNA contamination from random primer amplification.

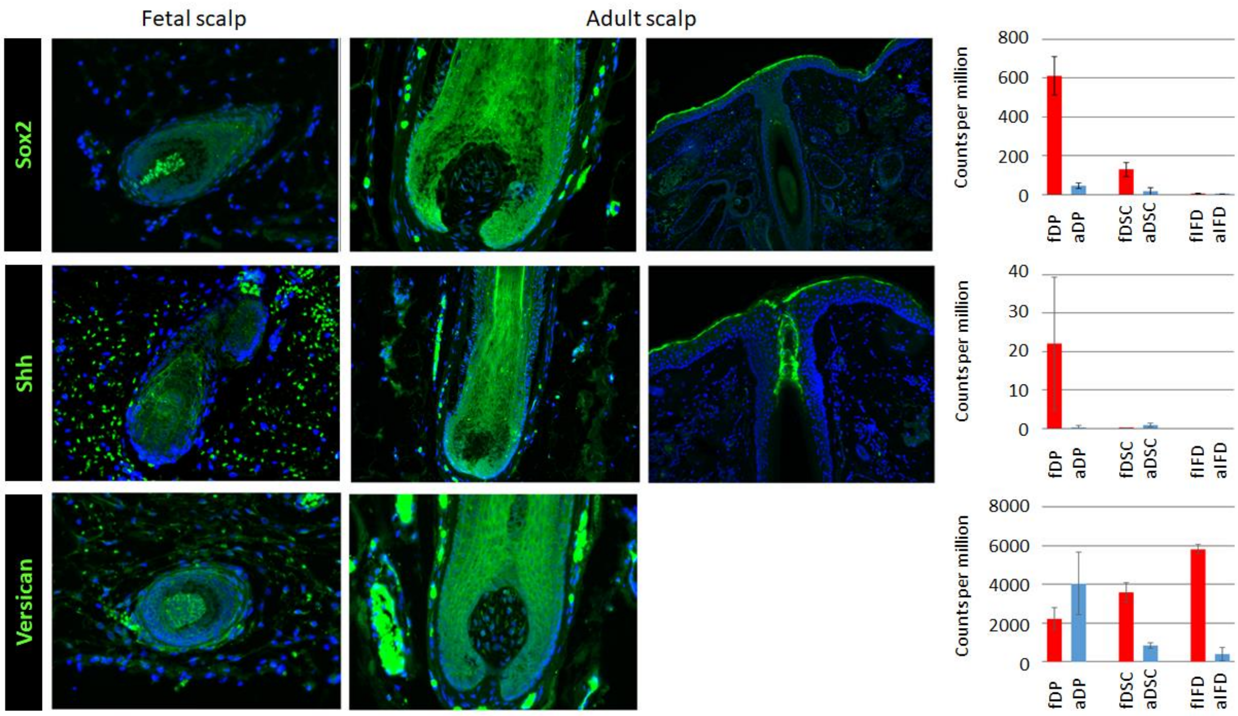

Figure S2. Validation of fetal RNAseq findings through immunofluorescent staining of human fetal and adult scalp with genes found to be present as a signature fetal DP gene, upregulated in fetal versus adult DP, and expressed at low levels or not expressed in DSC and IFD cells.

Table S1. Mapping analysis of RNA-Seq samples

Age	Tissue	Sample	Number of reads	Exonic reads	Intronic reads	Intergenic reads	5' bias	3' bias
Fetus	DP	ELW24	107,856,041	33.86%	41.00%	25.14%	0.12	0.31
		ELW26	111,369,332	25.06%	47.41%	27.53%	0.13	0.38
	DSC	ELW25	113,184,107	37.16%	35.76%	27.07%	0.20	0.37
		ELW27	109,670,397	34.16%	34.33%	31.51%	0.12	0.33
	IFD	ELW28	131,891,084	35.45%	30.18%	34.37%	0.10	0.38
		ELW29	107,847,992	39.58%	29.19%	31.23%	0.19	0.46
Adult	DP	ELW30	111,561,540	39.55%	26.00%	34.46%	0.18	0.36
		ELW33	119,706,161	36.53%	24.53%	38.94%	0.20	0.40

DP: Dermal papilla, DSC: Dermal sheath cup, IFD: Interfollicular dermis, 5' bias: The ratio between mean coverage at the 5' region (first 100 bp) and the whole transcript, 3' bias: The ratio between mean coverage at the 3' region (last 100 bp) and the whole transcript.

Table S2. The 121 signature fetal DP genes.

refGene	Log fold change (DP VS DSC)	P-Value (DP VS DSC)	False Discovery Rate (DP VS DSC)	Log fold change (DP VS IFD)	P-Value (DP VS IFD)	False Discovery Rate (DP VS IFD)
ADRB2	6.54615	3.23E-05	0.00203414	4.76653	8.42E-05	0.00255987
AGAP11	3.32507	0.00057252	0.0201036	3.58201	0.00015129	0.00409494
ANKRD29	2.68175	0.00068037	0.0228351	4.52187	3.41E-10	4.06E-08
ANO2	2.41348	0.00139328	0.0394272	3.68586	3.57E-06	0.000171108
APCDD1	4.50539	2.55E-10	5.76E-08	1.95969	0.00113299	0.0211019
APCDD1L	4.19585	1.52E-05	0.00105351	7.34579	2.70E-11	4.24E-09
AQP4	11.0845	0.00129758	0.0373041	11.0506	0.0005467	0.0119649
ARL9	9.41568	7.25E-14	3.49E-11	5.91547	9.81E-05	0.00288249
AVPR1A	2.86054	1.74E-06	0.000152482	2.30844	2.48E-05	0.000906468
BAMBI	7.08764	1.57E-13	7.10E-11	7.6088	2.76E-13	6.27E-11
BMP4	3.48698	1.45E-07	1.74E-05	3.44356	2.40E-08	2.00E-06
BMP7	4.98078	2.48E-11	7.02E-09	4.31737	1.15E-08	1.02E-06
BMP7-AS1	7.92973	0.00028237	0.0118447	12.1513	1.19E-06	6.78E-05
BOLA3-AS1	3.75967	0.0014081	0.0397686	3.82587	0.00046801	0.0105559
C3orf58	2.27779	0.0009782	0.0302431	2.23084	0.00060152	0.0130294
CACHD1	2.15273	0.00023974	0.0106132	1.72467	0.00162708	0.0282087
CACNA1C-AS1	8.32543	0.00061628	0.0211766	11.5911	2.39E-05	0.000880834
CALN1	5.82119	0.00121389	0.0356075	10.2097	2.09E-09	2.18E-07
CARNS1	5.01857	0.00108499	0.0324722	7.02025	2.58E-06	0.000129367
CDH24	2.71758	0.00160323	0.0436161	3.73879	0.000177	0.00467896
CDH8	6.40367	2.02E-08	2.91E-06	4.59175	7.87E-06	0.000348562
CENPW	4.68312	5.39E-06	0.00042048	3.12386	0.00019606	0.00510063
CNTNAP4	7.67689	3.25E-07	3.50E-05	14.115	7.09E-17	3.27E-14
COL23A1	5.29729	2.29E-09	4.17E-07	4.03955	6.22E-09	5.99E-07
CRYM	10.6653	4.64E-25	1.34E-21	3.62303	9.36E-07	5.55E-05
CSMD1	3.31928	7.91E-07	7.64E-05	5.00519	6.52E-14	1.66E-11
CSMD2	2.15857	0.00035045	0.0139602	6.00112	1.91E-17	9.71E-15
CYTL1	5.03706	1.35E-06	0.000121323	4.64111	5.59E-08	4.41E-06
DIO3	7.07949	1.05E-08	1.61E-06	6.1927	1.33E-08	1.16E-06

DPPA4	4.52052	5.52E-08	7.31E-06	3.3911	2.22E-06	0.000115141
DPY19L2	3.52969	1.26E-07	1.56E-05	2.36546	8.63E-05	0.00261319
EDN3	3.99855	5.52E-08	7.31E-06	4.04105	6.27E-07	3.86E-05
FABP7	5.21292	0.00107782	0.032339	6.12564	0.00063958	0.0135918
FGF10	5.48337	5.70E-06	0.000442258	3.62453	0.00017569	0.00465265
FGFR3	3.73377	9.11E-07	8.59E-05	5.15692	6.00E-13	1.28E-10
FRAS1	3.66835	4.13E-08	5.62E-06	6.31168	3.20E-16	1.31E-13
FST	3.04188	3.98E-07	4.22E-05	4.07344	1.94E-11	3.10E-09
FZD10	2.73343	0.00141477	0.0398789	2.94014	0.00076458	0.0155136
GFRA1	1.72586	0.00178606	0.0470372	3.46965	5.29E-10	6.10E-08
GNAL	4.5358	6.69E-05	0.00378693	3.28072	0.0015988	0.0279099
GNAZ	6.23482	2.20E-05	0.0014856	3.58968	0.00204909	0.0340182
GREB1	5.65366	2.15E-11	6.21E-09	4.18576	7.35E-08	5.64E-06
GRIA4	6.41082	5.58E-08	7.32E-06	6.44643	4.88E-06	0.000224962
GRID1	3.11045	5.37E-05	0.00319002	2.91433	0.00074149	0.0151912
HAND1	9.20218	0.00136461	0.0389211	11.4136	0.00024744	0.00618651
HIPK2	1.86898	0.00104616	0.0317164	1.98285	0.0002029	0.00525095
HS3ST3A1	3.97062	7.96E-05	0.0043832	10.1144	2.46E-13	5.66E-11
HS3ST3B1	2.87262	0.00059461	0.0206665	6.01512	2.33E-06	0.000119562
IFITM1	3.93547	0.0002536	0.0110069	2.67023	0.00042244	0.00963116
IGFBP3	3.2369	1.54E-05	0.00106634	4.1979	3.56E-08	2.89E-06
INHBA	2.41971	0.00036608	0.0142639	6.28743	3.38E-16	1.35E-13
ITGA8	2.17292	0.00043875	0.0163246	3.52475	5.11E-09	4.99E-07
KDM7A	2.78935	2.79E-05	0.00179916	2.52059	2.66E-05	0.000956601
LAMA3	4.175	3.51E-10	7.79E-08	4.6859	2.59E-15	9.33E-13
LAMB2P1	8.19861	2.20E-05	0.0014856	5.17995	0.00073016	0.0150427
LINC01139	5.37076	4.36E-11	1.19E-08	7.81926	5.85E-16	2.27E-13
LOC100289230	4.9847	0.00039994	0.0152293	4.34266	0.00080742	0.0162042
LOC100505715	4.90363	0.00066252	0.0223014	4.06414	0.00155897	0.0273115
LPL	3.01453	1.23E-05	0.000879223	6.78845	4.31E-14	1.12E-11
LRP10	4.01542	0.00129205	0.0373039	4.07381	0.00101695	0.019374
MAOA	7.03303	5.82E-06	0.00044907	5.12802	0.00133433	0.0242398
MAPK12	10.59	1.07E-07	1.36E-05	4.23212	0.00078913	0.0159023
MAPK4	8.17679	7.33E-07	7.25E-05	5.3449	0.00214033	0.0351774

MEGF11	13.7363	4.67E-13	1.93E-10	6.49781	2.15E-05	0.000803838
MGC12916	7.88248	3.77E-05	0.00232757	8.2779	1.65E-06	8.82E-05
MIAT	7.48003	5.58E-13	2.24E-10	5.01331	0.00022354	0.00566572
MMP1	11.4739	0.00023559	0.0104618	11.4257	7.28E-05	0.00225959
MPPED1	9.00113	0.00107521	0.032328	7.13055	0.0023315	0.0374232
MSX1	2.33344	0.00028315	0.0118447	4.46124	3.28E-10	3.93E-08
NCOA3	2.70228	2.92E-06	0.00024231	3.12509	1.77E-08	1.51E-06
NET1	4.13399	2.06E-09	3.92E-07	4.51411	3.14E-13	7.02E-11
NKD2	5.2695	3.77E-14	1.88E-11	4.62734	2.02E-12	3.93E-10
NOTUM	12.9486	2.34E-07	2.64E-05	5.79667	9.17E-05	0.00272248
NRG1	6.34625	0.00026704	0.0114698	7.18996	1.13E-07	8.33E-06
NRXN3	9.14677	2.13E-23	3.84E-20	7.37966	2.02E-22	2.49E-19
NSG1	2.90777	0.00175115	0.0462867	2.52695	0.00322192	0.047337
PAPPA2	4.14299	1.77E-05	0.00121211	3.32376	0.00227588	0.03665
PCDHB15	5.06509	8.40E-05	0.00458929	4.60819	0.0030113	0.044854
PCSK2	2.06221	0.00094035	0.0296311	4.63873	2.65E-05	0.000956601
PDE3A	2.51929	0.00010659	0.00545496	3.34374	6.43E-08	4.99E-06
PGM2L1	3.06007	2.44E-05	0.00160046	3.83824	1.91E-08	1.62E-06
PKNOX2	4.04791	1.51E-06	0.000133452	3.79497	5.20E-07	3.28E-05
PKP4	2.19225	0.00023515	0.0104618	2.86302	2.11E-07	1.49E-05
PMEL	4.99899	3.64E-12	1.32E-09	4.01976	8.23E-06	0.000361187
PPFIA2	5.40058	0.00012121	0.00601138	4.47915	0.00020717	0.00533901
PRLR	7.28498	1.52E-08	2.24E-06	6.91406	2.24E-17	1.10E-14
RABL6	2.80128	0.00068712	0.0230083	2.76389	0.00012175	0.00342381
RGCC	2.45201	7.45E-05	0.00415287	4.03557	4.86E-09	4.80E-07
RGS2	3.46534	2.92E-05	0.00186236	4.39134	2.73E-09	2.78E-07
RNF144B	4.57519	0.00063335	0.0216601	3.17825	0.00301338	0.044854
RPS6KA5	3.04697	0.00011141	0.00560251	3.02207	4.31E-05	0.0014685
RSPO3	7.17057	7.73E-23	1.24E-19	4.49732	3.04E-10	3.68E-08
RSPO4	5.46625	6.78E-12	2.33E-09	2.16408	0.00025029	0.00624714
SALL4	6.17977	0.00160478	0.0436161	7.2104	0.00022958	0.00580888
SCUBE1	8.28791	0.00044274	0.0164256	11.8205	8.67E-06	0.000377451
SEZ6L	3.83918	0.00151665	0.0419317	9.47715	1.02E-08	9.13E-07
SFRP1	2.45215	0.00014073	0.00677001	1.81676	0.00096673	0.018746

SHH	9.59247	0.00016582	0.00776984	11.7983	1.49E-05	0.000597027
SIM1	5.50407	0.00015339	0.00728205	3.28339	0.00073495	0.0150783
SLC27A6	4.77932	3.14E-09	5.46E-07	9.9766	3.86E-24	8.14E-21
SNED1	2.88249	0.00013067	0.0063708	2.5356	0.00021358	0.00545072
SOX2	2.24013	0.00012215	0.00603741	7.4757	2.89E-22	3.28E-19
SPATA31E1	11.3513	0.00029824	0.0122975	11.3073	8.94E-05	0.00267484
SPEF2	2.97565	0.00078484	0.0258015	2.40148	0.00038282	0.00883728
SPINT1	5.19874	0.0003327	0.0133676	8.25674	8.22E-07	4.97E-05
SPON1	3.11584	8.52E-08	1.10E-05	3.11747	7.95E-09	7.47E-07
TAF11	2.66955	0.00193487	0.0496433	2.17859	0.00238262	0.0380781
TBX3	5.12828	2.16E-09	4.06E-07	3.78296	5.61E-07	3.51E-05
TGFB2	1.90254	0.00095992	0.0298984	2.4889	5.69E-05	0.00185619
TMEM51-AS1	8.14251	0.00083002	0.0269193	9.6721	7.33E-05	0.00227149
TNFRSF19	3.53092	7.94E-07	7.64E-05	1.9042	0.00205018	0.0340182
TNK2	2.37459	0.00173183	0.0461141	2.46923	0.00334545	0.0484286
TPD52	3.91754	2.16E-07	2.49E-05	4.53291	1.29E-10	1.77E-08
TRIM2	3.42819	7.66E-09	1.23E-06	2.29017	1.93E-05	0.000735432
TRPM1	7.6545	8.56E-09	1.34E-06	5.21325	0.00048791	0.0109048
TRPS1	3.60268	3.46E-09	5.88E-07	2.75172	3.53E-07	2.34E-05
TYR	6.20018	1.38E-05	0.000968511	4.32036	8.41E-07	5.06E-05
WDR33	2.25303	0.0002705	0.0115499	1.89743	0.00139079	0.0249252
WNT5A	2.7833	9.30E-07	8.72E-05	4.89911	3.24E-11	4.92E-09
ZMYND8	2.57851	3.83E-05	0.0023514	2.22983	4.89E-05	0.0016328
ZSWIM5	3.63263	0.00023472	0.0104618	3.83963	9.05E-05	0.00269696

Table S3. The 822 differentially expressed genes between fetal and adult dermal papilla cells.

refGene	Log fold change (fetal DP vs. adult DP)	P-Value	False Discovery Rate
A2M	-6.73213	3.01E-12	1.03E-09
ABCB5	-8.95203	9.36E-05	0.00413583
ABCC9	-10.6408	8.64E-14	4.06E-11
ABCG1	-9.30923	3.83E-05	0.00201192
ABI3BP	5.43107	1.31E-09	2.56E-07
ACAN	6.10654	3.10E-06	0.00025612
ACKR1	6.08854	0.00135185	0.0303983
ACSL1	-7.18809	8.80E-08	1.11E-05
ACSL4	-2.8695	1.05E-05	0.00069567
ADAM23	-3.40035	0.00029716	0.0100531
ADAMTS18	7.97184	1.14E-05	0.00074591
ADAMTS5	3.8164	3.29E-06	0.00026925
ADAMTS6	3.19568	0.00164052	0.0349691
ADAMTSL1	6.46754	5.45E-10	1.20E-07
ADAMTSL4	4.11624	5.40E-06	0.0004076
ADCY1	6.86755	3.56E-07	3.76E-05
ADD2	-9.50587	0.00263263	0.0486405
ADGRA2	1.93316	0.00203979	0.0404218
ADGRB3	-4.22565	1.73E-09	3.21E-07
ADGRE5	3.06611	0.00201835	0.040209
ADGRG6	-4.45705	0.00041455	0.0128392
ADGRL1	3.23622	1.31E-06	0.00011614
ADGRL3	2.33095	0.00065077	0.0179032
ADGRL4	-9.19906	3.99E-05	0.00207539
ADGRV1	5.25361	6.81E-09	1.05E-06
ADM	-9.08748	2.05E-11	5.93E-09
ADRB2	2.67993	0.00254572	0.0473591
ADTRP	-10.7498	0.0001422	0.00571155
AFAP1L1	-5.1998	0.00085766	0.0221608
AFF2	2.97982	4.45E-05	0.002275
AGAP1	2.06655	0.00070483	0.0191105
AGAP2	5.86022	0.00111578	0.0262699
AGBL2	-7.00656	0.00253466	0.0472958
AGRN	2.77481	9.08E-05	0.00406089
AHR	-2.62453	4.69E-05	0.00233068
AIM1	-2.57445	2.05E-05	0.00119277
AK8	-6.21247	6.05E-05	0.00287662
AKAP12	-3.26788	2.15E-07	2.45E-05
ALDH2	-2.31048	0.00014528	0.00578856
ALDH3B2	-6.19908	0.00034314	0.010897
ALOX12P2	-6.56601	0.00127984	0.029128
ALOX15B	-14.825	9.37E-30	1.41E-25
ALPL	5.2163	3.96E-09	6.40E-07
AMD1	2.95476	1.65E-05	0.00099017
AMOT	3.76021	5.35E-06	0.0004076
AMOTL1	1.67744	0.00224124	0.0434956
ANK2	2.65368	2.25E-05	0.00128195
ANKRD29	4.98444	9.20E-11	2.30E-08
ANKRD50	2.89131	3.42E-06	0.00027756
ANO2	4.25259	1.15E-08	1.64E-06
ANXA1	-2.80767	0.00188572	0.0383962
APCDD1L	6.48113	3.71E-09	6.13E-07
APOD	-7.32498	1.84E-09	3.37E-07
APOL2	-3.49151	0.00032291	0.0105788
APOLD1	-6.43835	5.98E-11	1.57E-08
ARAP2	-3.97742	1.72E-07	2.00E-05
ARFGEF3	-7.60709	1.27E-05	0.00080779
ARHGAP23	1.97918	0.00105	0.025195
ARHGAP29	-2.01732	0.0013756	0.030801
ARHGAP6	-6.78462	0.00052961	0.0154171
ARHGDIB	-6.7469	7.03E-10	1.47E-07
ARHGEF28	-4.71594	7.78E-06	0.00053843
ARHGEF6	2.11106	0.00231062	0.0444972
ARL9	5.10577	1.59E-09	3.02E-07
ARMC5	3.6327	2.19E-05	0.0012623
ARPP21	-6.235	1.11E-05	0.00073456
ASIC2	11.0765	0.00024912	0.00872177
ASXL3	2.76466	0.000102	0.00439017
ATF3	-10.0762	6.21E-06	0.00045095
ATP1B2	3.61736	3.63E-06	0.00029175

ATP2C2	-8.79528	0.00087625	0.0222709
ATP8B4	-8.61057	5.60E-14	2.81E-11
ATRNL1	6.18768	8.78E-06	0.00059934
AVPR1A	4.58053	2.09E-09	3.69E-07
B3GAT3	3.89766	5.84E-05	0.00280236
BBOX1	-7.4795	0.00030187	0.0101215
BCAR3	2.38584	0.00255076	0.0473609
BCL2	2.50643	2.70E-05	0.00150649
BCL6	-3.26772	4.13E-06	0.000325
BHLHE40	-3.78878	1.41E-08	1.98E-06
BMP5	-7.72738	9.18E-07	8.78E-05
BNIPL	-8.79124	0.00025419	0.00882611
BOLA3-AS1	3.99361	0.00019127	0.00712933
BPIFC	-7.9253	0.00016138	0.00626385
BSN	5.35281	2.13E-12	7.58E-10
BST2	-9.93766	0.00151043	0.0327865
BTNL9	-8.67988	1.08E-07	1.31E-05
C10orf99	-10.544	0.00030413	0.0101292
C11orf95	3.314	1.19E-05	0.00077062
C17orf51	3.19329	0.0016717	0.0354671
C1orf106	-10.5401	0.00024547	0.00865539
C1orf116	-9.38997	1.42E-05	0.00088513
C1orf95	8.06367	2.32E-05	0.00131184
C3orf58	2.53233	6.40E-05	0.00302198
C8orf4	-8.13139	1.43E-05	0.00088513
CA2	-9.2287	3.36E-05	0.0017925
CA8	-10.0207	1.89E-07	2.17E-05
CACNA1C-AS1	6.25007	0.00029339	0.00997049
CACNA1G	4.3379	2.81E-07	3.15E-05
CACNA2D3-AS1	-10.5216	0.00025026	0.00872177
CALD1	-1.9322	0.00049711	0.0147155
CALML3	-7.21544	5.75E-07	5.76E-05
CALML3-AS1	-8.6868	1.28E-06	0.00011415
CAMK1G	-10.6518	0.0001574	0.00614116
CANT1	2.92623	0.00103415	0.02497
CAPN6	4.17287	5.12E-10	1.15E-07
CARD6	-10.9032	0.00011894	0.0050186
CARNS1	3.34556	0.00270771	0.0495402
CAV1	-2.08904	0.00172942	0.036231
CCBE1	4.93114	0.00025571	0.00883067
CCDC127	3.55455	0.00146345	0.0319048
CCDC65	4.326	0.00109376	0.0259137
CCDC69	-8.37786	2.02E-08	2.81E-06
CCK	-10.0005	0.00123743	0.0283345
CCNJL	4.4436	0.00197816	0.0396715
CCSER1	-6.30801	0.00150146	0.0326387
CCT5	-2.03288	0.00218393	0.0428261
CD163	-7.22615	0.00220981	0.043076
CD248	3.58858	7.39E-06	0.00051628
CD36	-9.72859	0.00203308	0.0403725
CD38	-11.5573	2.44E-06	0.00020384
CD74	2.69816	0.00082328	0.0216197
CD93	-3.6263	0.00267596	0.0493197
CDC42EP3	-4.04942	0.00055142	0.015747
CDCP2	-9.85487	0.00138436	0.0308066
CDH13	-9.42194	9.81E-10	2.02E-07
CDH5	-8.22549	4.82E-06	0.00036938
CDH8	3.34344	4.22E-05	0.00216974
CDKN1A	-5.08883	9.97E-09	1.48E-06
CDKN2B	-5.51965	8.35E-06	0.00057269
CEMIP	-2.55785	0.00015618	0.0061253
CENPW	3.11811	2.85E-05	0.00158207
CFAP57	-5.28578	0.00057397	0.0162672
CFH	-4.50686	0.00158982	0.0342131
CHD7	-2.89287	7.70E-05	0.00352767
CHST11	-2.39073	0.00257108	0.0476792
CHST15	-6.10027	8.78E-13	3.47E-10
CHSY3	4.85507	4.89E-05	0.00241716
CLDN5	-9.70686	0.00189904	0.038548
CLEC2B	-8.03364	5.37E-07	5.41E-05
CLIC5	-7.94631	0.00015589	0.0061253
CLMN	-4.02928	0.00017661	0.00666536
CLU	-7.21101	1.24E-08	1.75E-06
CNKSR1	4.81679	0.00057958	0.0163952
CNR1	-6.02814	0.00041864	0.0129389
CNTNAP2	5.65723	0.0008403	0.0219135
CNTNAP4	2.61993	0.00074547	0.019996
COL1A1	2.51	2.88E-05	0.0015896

COL1A2	2.25032	0.00033636	0.0107731
COL21A1	6.93637	5.36E-16	4.24E-13
COL6A6	8.60552	2.71E-16	2.39E-13
COL7A1	-2.38694	0.00191286	0.0387762
COLCA2	-9.6372	0.00192301	0.0389293
COLEC12	5.52381	6.35E-08	8.22E-06
COMP	-8.31802	3.61E-09	6.05E-07
CPA6	-6.34941	0.00018647	0.00700232
CPED1	4.35407	1.10E-06	0.00010093
CPM	-6.91242	3.90E-08	5.19E-06
CPXM1	4.44917	0.00042314	0.0130244
CRIP1	3.27279	0.00010681	0.00457094
CRTAC1	-6.65551	0.00160737	0.0344426
CRYM	3.99534	1.58E-09	3.02E-07
CSF3R	-11.149	9.07E-05	0.00406089
CSMD1	6.19743	1.14E-16	1.07E-13
CSMD2	5.43149	3.74E-15	2.25E-12
CXCR4	6.09446	4.19E-13	1.85E-10
CXorf36	-10.707	0.00013633	0.00551975
CXorf57	-6.85068	0.00141676	0.0312499
CYP27A1	-7.21994	0.00033561	0.0107731
CYR61	-4.30796	2.26E-05	0.00128195
CYSLTR2	-10.1898	0.00143197	0.0314461
CYTL1	5.94599	1.95E-11	5.85E-09
DAAM2	2.31983	7.49E-05	0.00346047
DACH1	-4.33947	0.00049767	0.0147155
DACT3	3.1579	3.23E-05	0.00175358
DAPL1	-5.66296	2.79E-14	1.44E-11
DBIL5P	-9.60899	0.00242138	0.0461569
DCHS1	2.91097	0.00044073	0.0134284
DCHS2	3.80524	0.00070672	0.0191274
DCLK1	-3.24951	5.27E-05	0.00257112
DCXR	-2.33502	0.00012403	0.00516059
DECR2	-8.48294	0.00076998	0.0205069
DENND2A	5.24226	2.58E-05	0.00144773
DENND3	-4.45469	4.18E-08	5.51E-06
DGKG	-9.89462	0.00122958	0.0282732
DGKH	1.70086	0.0024249	0.0461653
DIO2	-4.41923	2.91E-08	4.01E-06

DIO3	-2.47962	0.00019211	0.00714281
DKK1	8.27789	6.79E-10	1.44E-07
DLL4	-10.8918	9.00E-05	0.00406004
DNM3OS	2.93526	4.63E-05	0.00231837
DOCK11	-2.47784	0.00114633	0.0267791
DOCK8	-2.96502	1.63E-05	0.00097836
DPP6	-9.60147	3.05E-10	7.27E-08
DPPA4	3.08402	3.04E-05	0.00167105
DPY19L2	2.31936	0.00023411	0.00835298
DSC3	-2.62669	0.00014601	0.00580224
DSG1	-4.71176	5.59E-06	0.00042007
DSG3	-2.30581	0.00138923	0.0308693
DSG4	-7.88023	1.09E-09	2.17E-07
DSP	-2.91993	1.24E-05	0.00079779
DTX4	2.3162	0.00247214	0.0467682
DUSP1	-5.165	7.69E-05	0.00352767
DYRK1B	3.15518	0.00054994	0.015747
EBF1	2.74922	2.18E-06	0.00018432
EBF2	-10.0166	0.00102847	0.0248771
EDA2R	-9.87293	6.90E-07	6.77E-05
EDNRA	2.58444	7.74E-06	0.00053831
EDNRB	-4.0447	6.88E-06	0.00049213
EFHD1	-2.42521	0.00037338	0.0117578
EGFLAM	5.02192	1.51E-10	3.72E-08
EGFLAM-AS4	5.36856	0.00044923	0.0136193
EGFR	-2.22463	0.00163612	0.0349589
EGR1	-4.5076	3.84E-07	4.04E-05
EGR3	-3.39033	1.60E-05	0.00096602
ELF1	-2.18437	0.00143612	0.0314461
ELL2	-3.28629	0.00118435	0.0274962
ELMO1	2.66222	0.00013094	0.00535937
ELMOD1	5.23558	0.00039833	0.0124652
EMILIN3	2.8137	0.00081679	0.0215624
ENTPD1	-3.78315	1.86E-07	2.15E-05
EPAS1	-4.98263	5.10E-12	1.70E-09
EPB41L3	3.39237	0.0018186	0.0373697
EPHA6	7.1225	0.00031136	0.0102565
ERG	-6.55763	0.00032687	0.0106046
ESAM	-8.6504	1.14E-05	0.00074591

ETF1	-2.38385	0.0003076	0.0101569
ETV1	-2.58913	0.00030616	0.0101569
EXPH5	-3.07678	0.00086011	0.0221608
EYA4	8.57774	0.00020131	0.00738202
F2R	2.06153	0.00033845	0.0108166
F2RL2	2.29042	0.00030713	0.0101569
F2RL3	-9.47952	0.00246288	0.0467108
F3	-5.59594	1.04E-07	1.28E-05
FABP7	6.62282	9.05E-05	0.00406089
FAM107A	-10.0189	0.00134343	0.0302698
FAM110C	-11.7783	2.01E-06	0.00017225
FAM160A1	-5.83114	0.00100696	0.0245269
FAM168A	2.24846	0.00026669	0.00916701
FAM196B	-9.1704	0.00012103	0.00507827
FAM198B	-5.93597	1.40E-05	0.00087813
FAM43A	3.4991	9.67E-07	9.13E-05
FAM49A	-4.03284	0.0004745	0.0142548
FAM83H	-4.27343	0.00225712	0.0437473
FAT2	-3.55179	0.00094531	0.023316
FBLN1	-2.00736	0.00129327	0.0293447
FBN2	4.04576	3.37E-07	3.62E-05
FBXO32	-2.61112	4.50E-05	0.0022908
FER1L4	-10.2148	0.00065222	0.0179104
FFAR4	9.93664	0.00134412	0.0302698
FGD4	-6.44616	1.68E-11	5.15E-09
FGF1	-7.40234	2.13E-06	0.00018176
FGF10-AS1	2.27765	0.0010373	0.02497
FGF14	-9.06522	3.62E-09	6.05E-07
FGF18	-6.02188	0.00157678	0.0339811
FGF5	-10.4213	2.02E-09	3.61E-07
FGFR1	1.91517	0.00063091	0.0175499
FGFR3	2.84663	1.24E-05	0.00079779
FIGN	-5.42453	0.00102788	0.0248771
FILIP1	-9.11735	1.47E-12	5.38E-10
FILIP1L	2.28544	0.00183822	0.0377213
FKBP5	-5.30436	1.74E-10	4.22E-08
FLI1	1.82979	0.00250742	0.0469879
FLJ46066	6.74845	5.91E-06	0.00043699
FLRT3	4.01094	1.06E-08	1.54E-06

FLT1	-6.65124	7.88E-08	1.01E-05
FMN1	-2.4924	0.00088928	0.022518
FOS	-4.47637	0.00247037	0.0467682
FOSB	-8.14893	7.83E-06	0.0005393
FOXC1	-7.17889	0.00093297	0.0231639
FOXD1	9.85927	8.93E-09	1.35E-06
FOXK1	2.19551	0.0006486	0.0178765
FOXN1	-8.20409	6.35E-11	1.65E-08
FOXO1	-2.31425	0.00132983	0.0300382
FOXP1	-5.10099	2.17E-12	7.58E-10
FOXP2	-5.54119	1.30E-09	2.56E-07
FOXQ1	-7.01559	0.00041144	0.0127954
FREM1	2.16629	0.00060549	0.0170001
FRK	-10.5318	0.00023939	0.00850074
FRMPD1	-6.41868	0.00086506	0.0221652
FST	5.56319	6.45E-17	6.92E-14
FSTL1	1.93712	0.00094233	0.023316
FSTL5	4.13186	0.00085241	0.0221148
FZD10	2.66391	0.00021387	0.00779172
FZD2	5.06075	0.00045782	0.0138369
FZD6	-3.23245	0.00085749	0.0221608
GAB2	-2.73797	0.00020149	0.00738202
GALNS	3.6778	0.00025442	0.00882611
GALNT15	-5.00398	0.00054532	0.0157222
GALNT18	-8.214	0.00137822	0.0308066
GARNL3	3.29777	5.79E-05	0.00279678
GFRA1	2.5258	4.75E-05	0.00235234
GFRA2	6.07006	5.01E-05	0.00245849
GGT5	-4.82525	0.00021907	0.00791035
GGTA1P	6.84163	0.00168622	0.0355923
GIMAP4	-11.5002	1.69E-05	0.00100954
GJB2	-7.8732	1.57E-15	1.02E-12
GJB6	-2.93669	0.00107945	0.0256819
GLI1	2.78272	0.000859	0.0221608
GLIS2	4.00066	4.90E-08	6.40E-06
GLIS3	2.34246	0.00019743	0.00726866
GNG2	3.4983	0.00106683	0.0254766
GNL2	-2.89112	5.92E-05	0.00283336
GNRH2	10.1112	0.00168709	0.0355923

GPC3	3.52011	0.0006674	0.0181943
GPD2	-2.08376	0.00245652	0.046649
GPNMB	-3.31318	1.60E-05	0.00096602
GPRC5D	-10.0179	0.00103575	0.02497
GPX3	-9.62043	6.25E-19	8.76E-16
GRASP	2.83536	0.00138077	0.0308066
GREB1	2.59222	7.84E-05	0.00357835
GREM1	8.17176	2.19E-13	9.98E-11
GRHL1	-5.33064	9.14E-05	0.00407613
GRIA1	-4.23701	4.14E-05	0.0021434
GRID1	2.15329	0.00089943	0.0225767
GRID2IP	-10.0247	0.00096846	0.0237699
GRIK2	4.52176	0.00168239	0.0355923
GUCY1A3	-6.63448	0.00026898	0.00920358
HAAO	4.54421	1.13E-07	1.37E-05
HAP1	-11.7774	1.43E-19	2.38E-16
HBB	6.66052	0.00247639	0.0467897
HBEGF	-8.40737	5.65E-05	0.00274552
HBG2	6.09768	4.29E-06	0.00033182
HCLS1	-8.03208	0.00175992	0.0364631
HEPHL1	-7.71233	4.41E-09	7.05E-07
HERC5	-10.7704	0.0001134	0.00482522
HERC6	-8.37678	0.00061035	0.0171045
HES2	-6.13979	0.00087519	0.0222709
HEY2	2.80663	7.20E-06	0.00050901
HEYL	2.62222	0.00091887	0.0229347
HHIPL2	-7.95418	5.20E-07	5.31E-05
HID1	-5.78707	0.00236488	0.0451945
HIST1H1D	-2.0896	0.00050537	0.0148845
HIST1H3H	-9.87263	0.00215136	0.0422979
HLA-C	-4.66667	0.00199723	0.0399472
HLA-DPB1	6.96421	0.00218967	0.0428826
HMGCS1	-2.03322	0.00110567	0.026143
HOXC13	-3.03838	0.00024861	0.00872177
HOXD10	-10.0092	0.00116049	0.0270678
HS6ST1	3.1501	0.00121367	0.0280446
HSF2BP	-6.77941	0.0019493	0.0393026
HSPA2	-4.92605	5.94E-06	0.00043751
IFI27L2	3.2507	0.00026808	0.00919379
IFNLR1	-6.42203	0.00249577	0.0469786
IGDCC3	9.10013	6.21E-06	0.00045095
IGF2BP1	8.6298	1.06E-06	9.81E-05
IGF2BP3	5.07434	8.72E-05	0.00394735
IL11RA	2.67781	9.49E-05	0.00416641
IL1RAP	-4.8624	9.28E-05	0.00412191
INADL	-2.59695	0.00019579	0.00722604
INPP5D	-13.9251	3.70E-21	1.39E-17
IQGAP2	2.97252	0.00012603	0.00521518
ISYNA1	2.19746	0.00069018	0.0187812
ITGA6	-4.71932	1.04E-12	3.89E-10
ITGA8	2.67513	7.25E-06	0.00050901
ITGB4	-2.42735	0.00070279	0.0190898
ITGB5	2.15212	0.0016841	0.0355923
ITGBL1	-8.28873	8.46E-21	2.54E-17
ITM2C	2.94045	0.00015729	0.00614116
ITPR2	-2.19723	0.00230276	0.0444028
JAG2	-3.75871	0.00131923	0.0298887
JAK3	5.55626	1.64E-07	1.93E-05
JAM2	1.83837	0.00202452	0.0402786
JUN	-5.64607	1.29E-07	1.54E-05
KCNH8	7.39267	4.95E-13	2.07E-10
KCNJ8	-9.11496	9.45E-05	0.00416167
KCNK3	-10.3504	0.00041352	0.0128335
KCNN2	5.36739	0.00072506	0.0195532
KCNQ2	8.83694	2.21E-05	0.00126414
KCTD8	9.9398	0.00250088	0.0469879
KDR	-3.3087	6.88E-05	0.00323042
KIAA0040	-3.18826	7.39E-05	0.00343771
KIAA1024	-4.8895	0.00012823	0.0052771
KIF26B	4.19747	1.31E-05	0.00082668
KITLG	-2.68951	0.00033545	0.0107731
KLF3	1.83499	0.00136645	0.0306809
KLF9	-3.96644	3.25E-05	0.00175654
KLHL29	2.09875	0.00269118	0.0493729
KRT14	-3.54525	0.00052739	0.0153823
KRT28	2.45957	0.00215007	0.0422979
KRT31	-5.67352	8.93E-06	0.00060214
KRT35	-4.50679	1.42E-07	1.67E-05

KRT36	-9.91775	0.00140213	0.0311098
KRT5	-1.95546	0.002182	0.0428261
KRT85	-5.25835	8.85E-16	6.33E-13
KRTAP11-1	-6.96135	2.98E-08	4.07E-06
LAMA2	1.82142	0.00118226	0.0274902
LAMA3	4.10633	2.36E-11	6.70E-09
LAMB2P1	4.43882	0.00021741	0.00786905
LAMB3	-6.32514	2.15E-05	0.00124288
LAMC2	-5.62986	0.00056747	0.0161134
LAYN	-4.16443	0.00197678	0.0396715
LBH	-5.61969	0.00026033	0.00896872
LDHAL6B	-6.56042	0.00268595	0.0493729
LEFTY2	9.35584	9.52E-07	9.05E-05
LETM2	2.91331	0.00053086	0.0154234
LGR4	2.05125	0.00106618	0.0254766
LHFPL2	-2.65137	0.00173281	0.0362515
LHX2	2.93134	0.00065721	0.0180146
LIMS2	-7.05004	4.27E-05	0.00218887
LIN7A	-8.4552	0.00123287	0.0282732
LINC00473	-7.26663	0.00053307	0.0154283
LINC00629	10.2731	0.00055111	0.015747
LINC00648	-8.33651	0.0008845	0.0224426
LINC00954	5.36522	0.00017261	0.00658056
LINC01237	-10.1248	0.00089537	0.0225661
LIX1	5.50533	7.10E-06	0.00050566
LMF1	3.10672	0.00010928	0.00466315
LMO3	-4.81189	0.0009003	0.0225767
LMO4	2.53556	7.63E-05	0.00351648
LOC100130357	3.8729	0.00092165	0.0229587
LOC100233156	-10.0922	0.00083902	0.0219135
LOC100505715	4.75648	0.00053453	0.0154408
LOC100505912	-9.77373	0.00177383	0.0366197
LOC101054525	-9.47601	0.00249554	0.0469786
LOC101926975	-9.63076	0.00204352	0.0404422
LOC101928279	5.77524	0.00034228	0.0108928
LOC101928845	6.53839	0.00235684	0.0450981
LOC101929395	-9.61232	0.0020124	0.0401438
LOC101929524	-10.5553	0.0003035	0.0101292
LOC105378470	-14.3514	1.78E-25	8.93E-22

LOC148696	-10.6244	0.00032351	0.0105788
LOC284454	2.78902	0.00081591	0.0215624
LOC389834	-7.38585	0.00199682	0.0399472
LOC441204	-10.1735	0.00087004	0.0221882
LOC554206	4.93734	0.00205691	0.0406538
LOC643623	6.91766	0.00063998	0.0177274
LOC644285	2.34313	0.0007442	0.019996
LOC650226	-10.0314	0.00203462	0.0403725
LOC653602	-3.05833	0.00138323	0.0308066
LOC90768	8.99637	0.00015936	0.00620143
LRMP	-9.92966	0.00174316	0.0363163
LRP10	3.86523	0.00195798	0.0393849
LRRC16A	-2.76902	0.00025016	0.00872177
LRRC17	3.07863	0.00157068	0.0339471
LRRC32	-7.77355	9.16E-07	8.78E-05
LRRC7	10.1714	0.00086349	0.0221652
LRRTM3	7.092	1.42E-06	0.00012562
LSAMP	4.2871	0.00063419	0.0176083
LUZP2	5.12025	1.03E-09	2.10E-07
LYPD6B	-12.5557	3.28E-10	7.59E-08
MAFIP	-9.96786	0.00127759	0.0291209
MAGED2	2.78161	1.03E-05	0.0006853
MAMSTR	4.51463	0.00180753	0.0371931
MAOA	2.45215	0.00121543	0.0280446
MAP2	2.26013	0.00116393	0.027106
MAP7	-2.67886	0.00258716	0.0479182
MAPK12	3.40089	0.00174268	0.0363163
MASP1	-6.05719	0.00016411	0.00635342
MCTP2	-6.39953	0.00017047	0.00653238
ME3	2.70363	4.93E-05	0.00243018
MEAT6	-9.69503	0.00188343	0.0383962
MED12L	-6.47234	4.40E-07	4.55E-05
MEG3	1.78037	0.00175861	0.0364631
MEGF11	2.86717	0.00140502	0.031128
MEGF6	2.16268	0.00207353	0.0409283
MEST	7.13857	7.36E-16	5.53E-13
MEX3A	2.09408	0.00221101	0.043076
MFAP4	2.82711	4.62E-05	0.00231837
MGAT4A	-3.52686	0.00122943	0.0282732

MGP	-11.1758	2.99E-16	2.50E-13
MIAT	5.93344	4.58E-11	1.23E-08
MICALCL	-8.16561	0.00110716	0.026143
MINOS1	-4.51521	3.22E-05	0.00175358
MIR155HG	-4.27481	0.00011643	0.00494024
MIR34AHG	-7.33623	0.00092794	0.0230771
MIR6723	5.10929	6.50E-11	1.66E-08
MITF	-2.03709	0.00089046	0.022518
MLKL	-5.60176	0.00175859	0.0364631
MMP15	-11.1401	8.55E-05	0.00387944
MMP16	3.13771	3.95E-05	0.00206689
MMP2	3.18101	5.36E-07	5.41E-05
MN1	4.79711	3.50E-08	4.70E-06
MPZL2	-3.58485	0.00165216	0.0351516
MST1L	-4.14263	0.00251854	0.047112
MT1F	-8.89332	0.00013586	0.00551975
MT1M	-10.0529	0.00097297	0.0238029
MT1X	-7.24499	3.89E-09	6.35E-07
MT2A	-2.39722	5.76E-05	0.00278984
MT4	-2.7219	0.00221426	0.0430834
MTRNR2L6	-2.45743	0.00078989	0.0209824
MTRNR2L8	-2.46944	0.00024937	0.00872177
MURC	3.84625	0.00164125	0.0349691
MXRA5	6.13234	4.01E-15	2.32E-12
MXRA8	2.58725	0.00021423	0.00779172
MYCT1	-7.72373	0.00025573	0.00883067
MYH14	-4.18512	0.00012144	0.00508128
MYL3	3.75868	0.00036156	0.0114096
MYO1E	-2.61578	0.00049903	0.0147269
MYO1F	-3.45145	0.00126865	0.0289611
MYO6	-2.44989	0.00010274	0.00440926
MYRF	6.95592	3.62E-06	0.00029175
NACAD	4.8357	0.0005601	0.0159343
NAP1L5	-5.00469	0.00108055	0.0256819
NAPSA	7.17728	2.84E-06	0.00023603
NAV2	-2.16928	0.00016502	0.00635713
NCKAP5	-3.2999	5.72E-06	0.00042783
NCR3LG1	-3.57045	0.0011447	0.0267791
NDP	7.72844	6.94E-19	8.76E-16

NDST3	4.6725	0.00030304	0.0101292
NEAT1	-3.70355	1.14E-08	1.64E-06
NEGR1	-4.16252	0.00028113	0.00957576
NELL1	6.67568	4.45E-06	0.00034304
NET1	2.92855	2.16E-06	0.00018329
NFASC	-7.70786	4.19E-06	0.000326
NFIA	2.54547	0.00089876	0.0225767
NID2	2.30054	0.00250878	0.0469879
NIPAL4	-5.15269	1.63E-06	0.00014161
NKAIN4	11.2633	1.38E-05	0.00086987
NKD1	2.66111	3.27E-05	0.00175654
NKD2	4.11585	5.38E-06	0.0004076
NNAT	5.99871	4.50E-13	1.93E-10
NNMT	-9.43789	2.89E-07	3.20E-05
NOTCH1	2.71405	1.57E-05	0.00095466
NOTCH2	2.04393	0.00032396	0.0105788
NPTX2	3.6411	0.00170664	0.0358914
NPY1R	-7.81997	2.85E-05	0.00158207
NR1D1	-2.45096	0.00018794	0.00704005
NR4A1	-3.99071	0.00015293	0.00602924
NR5A2	-10.4959	0.000341	0.0108752
NRARP	-6.30395	0.00086619	0.0221652
NREP	2.76865	3.80E-06	0.00030168
NRG2	-6.43526	3.64E-05	0.00191793
NRG3	-7.91855	0.00270621	0.0495402
NRON	6.06031	0.00066258	0.0181285
NRXN3	2.32121	0.00140928	0.031155
NTM	-2.8524	0.00029911	0.0100966
NTN4	-10.6316	0.00023204	0.00831855
NTNG1	5.24812	9.83E-07	9.23E-05
NTNG2	7.83872	3.13E-11	8.70E-09
NTRK2	2.52455	1.56E-05	0.00095466
NTRK3	-3.46696	1.50E-06	0.00013119
NYNRIN	2.07097	0.00105399	0.0252503
OAS2	-6.83236	0.00091916	0.0229347
OBSL1	2.02668	0.0015948	0.0342221
ODAM	-9.90495	0.00132153	0.0298956
OGDHL	-9.56843	0.00233054	0.0447661
OGFRL1	-2.55106	0.00152378	0.0330285

OLFML1	2.58471	0.00063064	0.0175499
OMD	3.12772	0.00011791	0.00498896
OR2M4	-9.82079	0.00234666	0.0449606
OSMR	-2.19332	0.00159337	0.0342221
OVOL1	-5.81376	0.00050775	0.0149256
PAEP	6.82767	0.00194715	0.0393026
PAG1	-2.40303	0.00061218	0.0171239
PAK3	-10.1455	0.00073753	0.0198539
PALMD	-5.34979	0.00049242	0.014676
PAM	-1.87454	0.00195862	0.0393849
PAQR6	3.79646	0.00016505	0.00635713
PARM1	-3.30618	5.99E-05	0.00285405
PARP8	-2.39682	0.00113708	0.026646
PAWR	-4.73793	9.98E-05	0.00431917
PCDH17	-7.75818	0.00049448	0.0147082
PCDHAC2	6.4345	1.02E-06	9.41E-05
PCDHB11	3.32797	8.41E-05	0.00383017
PCDHB15	3.75711	0.00033494	0.0107731
PCDHGA6	3.45141	0.00049563	0.0147133
PCDHGB8P	3.28353	0.00014221	0.00571155
PCK1	-7.98315	0.00014922	0.0059139
PCNX2	-2.97088	9.73E-05	0.00424714
PCOLCE	2.64548	1.97E-05	0.00115291
PCSK2	3.22434	3.46E-07	3.69E-05
PCSK6	-10.7104	0.00013083	0.00535937
PDE2A	-9.62558	5.22E-09	8.17E-07
PDE3A	2.0883	0.00111801	0.0262811
PDE4B	-9.10512	5.81E-07	5.78E-05
PDE7B	-6.73781	0.00022259	0.00801807
PDLIM5	-3.38242	0.00079063	0.0209824
PDZD4	3.93769	0.00085244	0.0221148
PEG10	2.73809	0.00033637	0.0107731
PGF	-11.1866	3.96E-05	0.00206689
PGM2L1	2.38539	0.00082325	0.0216197
PGR	-7.68332	0.00012316	0.00513862
PIANP	2.3925	0.00059016	0.0166319
PIK3R1	-2.68318	1.27E-05	0.00080779
PIRT	-10.7485	0.00012032	0.00506244
PITX2	6.1146	0.00064084	0.0177274

PKLR	-9.74865	0.00169685	0.0357482
PKNOX2	5.38749	1.99E-11	5.85E-09
PLCXD3	-8.33061	0.00222363	0.0432098
PLEKHN1	-8.14751	0.00141038	0.031155
PLVAP	-8.16948	1.23E-06	0.0001107
PLXDC2	-2.56456	0.00013862	0.00559748
PLXNA2	-2.93873	0.00017438	0.00663136
PMFBP1	-13.2186	6.91E-12	2.21E-09
PNPLA7	-5.83437	0.00040261	0.0125731
PODN	3.68024	0.00100746	0.0245269
POSTN	-7.39641	0.00177065	0.0366197
PPL	-2.28405	0.00177479	0.0366197
PPP1R15A	-5.59075	1.50E-06	0.00013119
PPP1R9A	4.51167	0.00229143	0.044241
PRDM8	-8.50082	0.00062989	0.0175499
PREX1	1.95645	0.00086184	0.0221652
PRICKLE2	2.17497	0.00058535	0.0165272
PRICKLE2-AS1	4.2489	0.00254635	0.0473591
PRKCH	-4.64266	0.00015156	0.00599085
PRKCZ	-10.2809	0.000486	0.0145133
PRLR	4.98699	9.60E-13	3.70E-10
PROK1	-12.2617	4.20E-17	4.86E-14
PROKR2	-10.4103	1.04E-08	1.53E-06
PRR5	-6.18325	0.00236813	0.0451991
PRRT3	3.07299	0.00148191	0.0322606
PRRX1	2.15018	0.00085128	0.0221148
PRSS12	-6.03327	8.13E-07	7.93E-05
PRSS16	-10.2869	0.00055431	0.0157994
PRUNE2	-10.62	2.70E-20	5.79E-17
PSMD5-AS1	4.39105	0.00265423	0.0489794
PSTPIP2	2.87818	0.00167129	0.0354671
PTCH1	2.29274	0.00075875	0.0203158
PTCH2	2.6019	7.44E-05	0.00345053
PTCHD1	11.8602	1.19E-05	0.00077062
PTCHD4	-9.87588	0.00170843	0.0358914
PTGDS	-7.83253	0.00268186	0.0493679
PTGES	-3.16459	0.00019399	0.0071947
PTGIS	4.01885	0.00044972	0.0136193
PTH1R	2.714	5.14E-05	0.00251485

PTP4A1	-3.35869	4.13E-07	4.31E-05
PTPN3	-3.24554	0.0012044	0.0279186
PTPRB	-4.93617	4.18E-06	0.000326
PTPRE	-5.99211	8.94E-06	0.00060214
PTPRT	4.56836	0.0007083	0.0191356
PURG	4.05947	0.00059613	0.0167688
PVRL3	2.14812	0.00220372	0.0430572
PYGO1	2.10216	0.00051917	0.0152314
RAB30	2.35395	0.00104265	0.0250587
RAB3C	-11.1422	3.37E-05	0.0017925
RAPGEF3	-5.55408	7.22E-05	0.00337005
RAPGEF4	-3.25924	9.70E-05	0.00424714
RASD1	-9.72893	5.60E-13	2.27E-10
RASL12	-9.49575	0.00243511	0.046301
RBP4	9.32783	8.45E-20	1.59E-16
REM1	-9.64677	0.00193342	0.0390874
RET	5.22989	0.00020627	0.00753872
RFTN2	2.33116	9.35E-05	0.00413583
RGCC	3.27431	9.99E-07	9.32E-05
RGL3	-12.3781	5.22E-09	8.17E-07
RGS5	-6.50052	7.00E-19	8.76E-16
RHBDF2	-6.86255	0.00083856	0.0219135
RIMBP2	3.90007	0.00094289	0.023316
RIMS2	-6.26832	0.00017243	0.00658056
RMST	-8.42331	0.00052158	0.0152722
RNASE1	-7.06278	0.00145976	0.0318707
RNF217	-3.22777	6.20E-07	6.12E-05
ROBO2	3.20348	9.67E-08	1.20E-05
RPRM	9.93744	0.00112662	0.0264421
RSPO2	-8.91782	9.87E-16	6.74E-13
RSPO3	3.73968	9.30E-08	1.16E-05
RSPO4	-1.82328	0.00157459	0.0339811
RTN4RL2	6.98591	0.0002955	0.0100197
RUNX1T1	2.02477	0.00038173	0.0119957
RUNX3	2.05776	0.00080763	0.0213957
S100A14	-3.70691	4.67E-07	4.81E-05
S100A16	-2.62269	0.00097053	0.0237818
S1PR3	-4.05464	0.00110866	0.026143
SAT1	-2.75328	3.30E-06	0.00026925

SATB1	2.62739	7.07E-05	0.00330611
SBSPON	-4.74167	5.94E-10	1.29E-07
SCNN1A	-10.0877	0.00107069	0.0255283
SCUBE1	4.84652	0.0017419	0.0363163
SCUBE3	2.32616	4.56E-05	0.0023091
SEMA5A	-2.99058	1.96E-05	0.00115291
SERPINB13	-9.7845	3.15E-10	7.39E-08
SERPINB5	-3.67271	2.12E-05	0.00122853
SERPINB8	-5.61013	7.37E-09	1.13E-06
SERPINE1	-9.98843	7.93E-17	7.94E-14
SERPINH1	2.0058	0.00145199	0.0317471
SEZ6L	3.26061	0.00156099	0.0337863
SFN	-1.88774	0.00172383	0.0361644
SGCE	2.19077	0.00095568	0.0234946
SGF29	3.46082	0.00046943	0.014138
SGIP1	-9.981	3.26E-28	2.45E-24
SGMS2	-5.47134	1.26E-05	0.00080372
SH3D19	-2.0602	0.00090721	0.0227119
SHB	2.63164	5.81E-05	0.00279678
SHH	6.08567	0.00108674	0.0257882
SIDT2	2.30444	0.00081946	0.0215948
SLC11A1	-7.51582	0.00054829	0.015747
SLC16A6	-7.30168	0.0001432	0.00573605
SLC27A6	2.6545	1.97E-05	0.00115291
SLC2A9	-9.40172	2.21E-05	0.00126414
SLC35E2	2.48888	0.00086858	0.0221882
SLC35F3	-10.578	0.00030766	0.0101569
SLC39A11	-7.89745	1.28E-07	1.54E-05
SLC45A4	-2.86403	0.00101837	0.0247524
SLC46A3	-10.4791	0.00028102	0.00957576
SLC6A15	-3.4778	0.00123103	0.0282732
SLC7A10	-10.386	0.00044412	0.0135044
SLC7A11	-7.93536	1.94E-14	1.04E-11
SLC7A11-AS1	-7.89701	1.80E-05	0.00106669
SLC7A8	-2.50608	0.00048272	0.0144441
SLCO1C1	3.55764	0.00259294	0.0479662
SLCO4A1	-10.0494	0.0009493	0.0233762
SLIT3	3.07191	2.74E-07	3.09E-05
SNAI1	3.30938	6.87E-05	0.00323042

SNCA	-3.50971	0.00187745	0.0383962
SNHG8	-3.0781	0.00175579	0.0364631
SNTB1	-4.65691	2.42E-09	4.18E-07
SOBP	2.89295	0.00013381	0.00546182
SOCS3	3.22629	9.86E-05	0.00428124
SOSTDC1	-2.73892	1.04E-05	0.00069171
SOWAHC	-2.77552	0.00231391	0.0445036
SOX11	4.9991	2.50E-05	0.00140601
SOX2	3.74451	3.44E-09	5.87E-07
SOX2-OT	4.84777	0.00042884	0.013144
SOX8	6.17009	3.78E-06	0.00030168
SOX9	-5.8639	0.00142399	0.0313174
SP100	-3.45275	5.89E-06	0.00043699
SP140L	-9.73222	0.00234331	0.0449539
SPARCL1	-4.51404	1.66E-11	5.15E-09
SPATA31E1	11.0911	3.41E-05	0.00180875
SPON1	2.53868	6.31E-05	0.00299115
SQRDL	-10.4032	0.00038478	0.0120664
SRD5A2	-10.8632	3.84E-11	1.05E-08
SRGN	-11.7496	1.68E-06	0.00014482
SRPX	3.85851	2.98E-07	3.24E-05
SRRM4	6.5129	0.00076713	0.0204672
SST	-11.0149	1.99E-09	3.61E-07
SSTR2	-8.99345	2.80E-15	1.75E-12
ST14	-3.05278	0.00043657	0.0133288
ST6GALNAC5	-11.6713	6.19E-06	0.00045095
STAB1	-7.13536	0.00269199	0.0493729
STAC2	-5.22097	0.00187311	0.0383847
STAG3	4.65423	1.44E-05	0.0008929
STIM2	-2.08058	0.00188645	0.0383962
STK10	3.47799	0.00160977	0.0344449
STMN3	3.01222	1.71E-05	0.00101502
STON2	-2.63998	0.00034418	0.0109069
STRA6	-8.93338	0.00016896	0.00649084
STX18	2.94243	3.91E-06	0.00030934
STX18-AS1	4.29155	0.0003267	0.0106046
SUSD2	-5.96638	0.00250874	0.0469879
SYCP3	5.08205	0.00042965	0.013144
SYNJ2	-2.28587	0.00042896	0.013144
SYT1	-8.96505	0.00040731	0.0126933
TACSTD2	-9.05201	8.04E-14	3.90E-11
TBX18	3.44344	8.35E-07	8.10E-05
TBX2	-10.2356	0.00064318	0.0177595
TBX3	2.61829	0.00024273	0.00857888
TEAD2	1.95787	0.00189154	0.0384476
TENM1	-9.98258	2.19E-20	5.49E-17
TENM2	-3.21256	2.28E-06	0.00019097
TENM3	4.72673	2.19E-09	3.82E-07
TENM4	4.02178	3.55E-05	0.00187573
TESC	-9.54573	0.00248153	0.046828
TET1	1.85675	0.00209273	0.0412532
TFPI	-7.62153	4.68E-05	0.00233068
TGM2	-8.83211	0.00019091	0.00712933
THBD	3.92269	1.67E-09	3.13E-07
THBS1	-6.20019	4.84E-10	1.10E-07
THSD4	2.5073	3.17E-05	0.00173298
THSD4-AS2	6.30577	0.00031518	0.0103596
TIGD1	2.95242	0.00120644	0.0279229
TIGD2	4.57589	0.00252652	0.0472026
TIGIT	6.67909	0.00022948	0.0082463
TIMP3	-1.84129	0.00226411	0.0438263
TLE4	-2.16767	0.00137591	0.030801
TLR2	-5.18439	1.22E-06	0.00011058
TM6SF2	5.99244	0.00188142	0.0383962
TMCC3	-5.09768	0.00014474	0.0057821
TMEM119	4.24698	0.00012752	0.00526236
TMEM132C	9.30827	6.52E-12	2.13E-09
TMEM145	10.3997	0.00048148	0.0144357
TMEM171	10.9367	0.00030046	0.0101193
TMEM184A	-7.25134	0.00129147	0.0293447
TMEM198B	2.40125	0.0004221	0.0130193
TMEM51-AS1	7.67281	0.00024142	0.00855278
TMEM92	-6.6947	0.00098564	0.0240736
TMEM92-AS1	-10.6023	0.00023739	0.00844987
TNC	-3.14656	1.47E-05	0.00090206
TNFSF10	-8.80567	0.00021615	0.00784229
TNK2	2.1172	0.00179413	0.036968
TNS4	-2.22621	0.00018252	0.00687123

TP73	-7.11447	0.00076014	0.0203168
TPBG	2.40207	0.00052476	0.0153354
TRERF1	3.61563	2.85E-07	3.17E-05
TRIB1	-3.85863	3.27E-05	0.00175654
TRIB3	-10.216	0.00102242	0.0248107
TRIM2	2.03762	0.0003264	0.0106046
TRIM29	-2.03454	0.00055044	0.015747
TRIM9	-7.4889	0.00012469	0.00517389
TRNP1	-7.95479	0.00271413	0.0495973
TRPC6	-9.49284	7.25E-06	0.00050901
TSC22D3	-2.39145	0.00032792	0.0106157
TSHR	-6.49501	3.12E-05	0.00171265
TSKU	2.37877	0.00066431	0.0181428
TSPAN18	2.69094	9.29E-06	0.00062297
TTC28	2.05109	0.00228173	0.0441105
TTYH1	6.99171	8.94E-06	0.00060214
TUBA4A	-3.16252	0.00046967	0.014138
TUSC5	9.98374	2.94E-07	3.23E-05
UNC5D	-5.92592	3.31E-07	3.58E-05
USP49	2.14633	0.0020071	0.0400912
UTS2B	-9.98991	0.00142287	0.0313174
VCAM1	3.42459	1.49E-05	0.00091254
VDR	-6.39338	9.90E-09	1.48E-06
VIT	-3.35818	4.57E-05	0.0023091
VSNL1	-5.3405	8.33E-08	1.06E-05

VWF	-9.7445	6.66E-10	1.43E-07
WDR86	3.50019	0.00010081	0.00435122
WEE1	-2.81293	9.86E-05	0.00428124
WIF1	3.35008	6.59E-06	0.00047562
WISP1	-8.54663	1.63E-14	9.06E-12
WNT5A	2.63288	4.61E-05	0.00231837
XKR5	6.87206	0.00089483	0.0225661
ZBTB16	-8.36946	6.63E-06	0.00047624
ZBTB7B	-4.57206	0.00030169	0.0101215
ZDHHC23	-4.99499	0.00053188	0.0154234
ZEB2	1.93102	0.0008341	0.0218657
ZFHX3	-2.27452	0.00023272	0.00832306
ZFP36	-2.86938	0.00143593	0.0314461
ZNF154	2.7074	0.00017487	0.00663313
ZNF185	-3.05562	1.20E-06	0.00010911
ZNF257	4.15227	0.00126556	0.0289346
ZNF275	1.98539	0.00220432	0.0430572
ZNF366	-8.62389	0.00035967	0.011374
ZNF385A	-3.14776	4.16E-05	0.00214801
ZNF503-AS2	5.09733	0.00017568	0.00664692
ZNF521	2.39157	0.00019458	0.00719909
ZNF704	2.21661	0.00013616	0.00551975
ZNF727	-6.77157	0.00094376	0.023316
ZNF750	-4.9152	3.32E-08	4.49E-06
ZNHIT6	-1.95551	0.00254751	0.0473591
